# Pseudobulk with proper offsets has the same statistical properties as generalized linear mixed models in single-cell case-control studies

**DOI:** 10.1093/bioinformatics/btae498

**Published:** 2024-08-08

**Authors:** Hanbin Lee, Buhm Han

**Affiliations:** Department of Medicine, Seoul National University College of Medicine, Seoul, 03080, Republic of Korea; Department of Statistics, University of Michigan, Ann Arbor, 48109, United States; Department of Medicine, Seoul National University College of Medicine, Seoul, 03080, Republic of Korea; Department of Biomedical Sciences, BK21 Plus Biomedical Science Project, Seoul National University College of Medicine, Seoul, 03080, Republic of Korea; Interdisciplinary Program in Bioengineering, Seoul National University, Seoul, 03080, Republic of Korea

## Abstract

**Motivation:**

Generalized linear mixed models (GLMMs), such as the negative-binomial or Poisson linear mixed model, are widely applied to single-cell RNA sequencing data to compare transcript expression between different conditions determined at the subject level. However, the model is computationally intensive, and its relative statistical performance to pseudobulk approaches is poorly understood.

**Results:**

We propose offset-pseudobulk as a lightweight alternative to GLMMs. We prove that a count-based pseudobulk equipped with a proper offset variable has the same statistical properties as GLMMs in terms of both point estimates and standard errors. We confirm our findings using simulations based on real data. Offset-pseudobulk is substantially faster (>×10) and numerically more stable than GLMMs.

**Availability and implementation:**

Offset pseudobulk can be easily implemented in any generalized linear model software by tweaking a few options. The codes can be found at https://github.com/hanbin973/pseudobulk_is_mm.

## 1 Introduction

Modern single-cell RNA sequencing (scRNA-seq) data involves cells from multiple subjects and donors (Yazar *et al.* 2022, Li *et al.* 2023). Researchers have found that when comparing RNA expression levels across subjects in different conditions, called the case-control differential gene expression (DGE) analysis, one should take subject-specific variability into account. Otherwise, the false discovery rate (FDR) can increase substantially (Squair *et al.* 2021, Zimmerman *et al.* 2021). Generalized linear mixed models (GLMMs), such as Poisson generalized linear mixed model (PGLMM) and negative-binomial generalized linear mixed model (NBGLMM), are widely used to address this problem. Nevertheless, GLMMs are computationally burdensome, making them intractable for large datasets (He *et al.* 2021, Nathan *et al.* 2021).

The computational burden stems from the subject-specific random intercept that absorbs the subject-level variability to produce calibrated *P*-values (Stroup 2016). While generalized linear models (GLMs) for count data have closed-form likelihoods, GLMM likelihoods do not enjoy this property (Bates *et al.* 2015). As a result, the likelihood computation requires numerical integration, slowing down the fitting process. Furthermore, all GLMM methods suffer from numerical instability due to the non-convex loss function of GLMMs, especially in low-expression transcripts.

A lightweight alternative to GLMM is the pseudobulk approach. Pseudobulk methods aggregate single-cell counts into one value per subject, mimicking bulk RNA-seq data. This aggregation effectively removes the need to model within-subject variability, dramatically improving computational speed. It is several times faster than the fastest mixed model. For example, NEBULA, a software tailored for large scRNA-seq data, uses an approximate likelihood, assuming that the number of cells per subject is sufficiently large (He *et al.* 2021). However, pseudobulk is often seen as an inferior heuristic to GLMM due to the aggregation step, sacrificing full probabilistic modeling for efficiency (Zimmerman *et al.* 2021).

Many papers have attempted to compare GLMMs and pseudobulk, showing mixed results on their relative performance (Squair *et al.* 2021, Zimmerman *et al.* 2021, Junttila *et al.* 2022, Murphy and Skene 2022). In contrast to the common belief that pseudobulk and GLMMs are very different methods, we theoretically prove that a simple pseudobulk approach with a carefully selected offset covariate has almost the same statistical properties, such as point estimates and standard errors, as GLMMs in the case-control setting. We call this approach the offset-pseudobulk.

Along with its asymptotic equivalence with GLMMs, offset-pseudobulk has two favorable properties compared to GLMM: It is (i) highly scalable as the runtime only depends on the number of subjects and not the cells, and (ii) numerically more stable than GLMM. In sum, we provide a theoretical foundation that pseudobulk methods, with proper offsets, can safely replace the use of computationally more intensive GLMMs in case-control DGE analysis.

## 2 Materials and methods

### 2.1 GLMs and linear mixed models

Unique molecular identifier (UMI) scRNA-seq counts are positive integers. Count-based generalized linear and mixed models take this into account by modeling them as Poisson or negative-binomial distributions. They fit a regression with a log-link function using the raw UMI counts. In DGE analysis, the parameter of interest is the log-fold change (logFC), which is the log ratio of the mean expression of the two conditions. Thanks to the log-link function, the regression coefficient of the condition variable immediately has a logFC interpretation.

Normalization is a crucial step in DGE analysis. In count-based models, size factors that quantify the overall abundance of transcripts are first computed (Lun *et al.* 2016). The inclusion of the size factor as an offset with a coefficient of 1 serves as a normalization.

In case-control DGE, pseudobulk sums up the counts of each transcript within each subject. This aggregation is called pseudobulk because the observations at a cell level are collapsed into a subject-level observation, which is similar to traditional bulk sequencing data. Many tools supply the aggregated counts to GLMs to obtain logFC.

Count-based GLMs are implemented in DESeq2 (Love *et al.* 2014), edgeR (Robinson *et al.* 2010), and glmGamPoi (Ahlmann-Eltze and Huber 2021). They first estimate the regression coefficients using the Poisson likelihood. Next, the dispersion parameter of a negative-binomial likelihood is estimated with the regression coefficients from the previous step fixed. Finally, small corrections are made to the initial regression estimates using the newly obtained dispersion estimates. As all three software implement the same regression model, we used glmGamPoi for our analysis.

Poisson and negative-binomial GLMM are famous alternatives to pseudobulk. GLMMs directly take cell-level observations. In case-control DGE, they include a random intercept for each subject to account for the covariation of transcript abundance within a subject. The objective function of GLMMs does not have a closed-form expression because of the random intercept. Hence, optimizing the likelihood is computationally burdensome, especially in large datasets. NEBULA alleviates this burden by approximating the likelihood (He *et al.* 2021). Computing the approximate likelihood is substantially easier because it has a closed-form expression. Nevertheless, pseudobulk remains several times faster than GLMMs in runtime.

### 2.2 From cell-based GLMMs to subject-level GLMs

Here, we introduce offset-pseudobulk as an alternative to fitting GLMMs for case-control DGE. It is often believed that GLMMs are more powerful than pseudobulk because they preserve cell-level information (Zimmerman *et al.* 2021). However, we show that no information is lost in pseudobulk by aggregating the counts, i.e. the power of the test remains intact (Murphy and Skene 2022). Furthermore, GLMM and pseudobulk produce very close estimates, as we show mathematically in the following paragraphs.

We prove that a count-based GLMM applied to cells can be expressed as a count-based GLM applied to subjects. The following derivation shows how to convert a regression on cells to a regression on subjects.

A typical regression specification written in R formula-like syntax of case-control DGE is

(1) $$Y_{\mathrm{ijk}}∼\log\;s_{j}+\beta_{0k}+X_{i}\beta_{1k}+\alpha_{i},\;$$

where i is the index for subjects, j is the index for cells, and k is the index for transcripts. $Y_{\mathrm{ijk}}$ is the transcript count, $s_{j}$ is the size (normalization) factor of cell j. $\beta_{0k}$ is the intercept and $\beta_{1k}$ is the coefficient of case-control label $X_{i}$ of subject i. $\alpha_{i}$ is the subject-specific random effect, which models the within-subject correlation of cells in GLMM.

Assuming Poisson regression for count data, the R formula corresponds to a conditional mean relationship

(2) $$E[Y_{\mathrm{ijk}}\;|\;X_{i},s_{j},\alpha_{i}]=\exp(\log\;s_{j}+\beta_{0k}+X_{i}\beta_{1k}+\alpha_{i})=\;s_{j}e\mathrm{xp}\;(\beta_{0k}+X_{i}\beta_{1k}+\alpha_{i}).$$

Averaging Equation (2) over $\alpha_{i}$ gives

(3) $$E[Y_{\mathrm{ijk}}\left|\;X_{i},s_{j}\right]=E\left[E[Y_{\mathrm{ijk}}\;|X_{i},\;s_{j},\alpha_{i}\right]\left|X_{i},s_{j}\right]=\exp\left(\log\;s_{j}+\beta_{0k}+X_{i}\beta_{1k}\right)E\left[\exp\;\alpha_{i}\right]=\exp\left(\log\;s_{j}+{\beta '}_{0k}+X_{i}\beta_{1k}\right),$$

where ${\beta '}_{0k}=\beta_{0k}+\log E\left[\exp\;\alpha_{i}\right].$

Due to the property of the conditional expectation (Durrett 2010), the residual $Y_{i}-\;E\left[Y_{\mathrm{ijk}}|X_{i},\;s_{j}\right]$ is uncorrelated with the case-control status ${\left(1,\;X_{i}\right)}^{T}$;

(4) $$0=E\left[\left[\begin{array}{c} 1\\ X_{i} \end{array}\right]\left(Y_{\mathrm{ijk}}-\;E\left[Y_{\mathrm{ijk}}|X_{i},\;s_{j}\right]\right)\right]=E\left[\;\left[\begin{array}{c} 1\\ X_{i} \end{array}\right]\left(Y_{\mathrm{ijk}}-s_{j}\exp\left({\beta '}_{0k}+X_{i}\beta_{1k}\right)\right)\right].$$

The solution for GLMM satisfies Equation (4). The inverse is also true because Equation (4) consists of derivatives of convex functions; the unique solution for Equation (4) will correspond to the solution for GLMM. The solution $({\beta^{'}}_{0k}$, $\beta_{1k}$) for Equation (4), though, does not fix a solution for GLMM, as we cannot decompose ${\beta '}_{0k}$ into $\beta_{0k}$ and $\log E\left[\exp\alpha_{i}\right]$. However, $\beta_{1k}$ for Equation (4) is exactly $\beta_{1k}$ for GLMM, which is the parameter of our interest. Thus, if we solve Equation (4), in terms of $\beta_{1k}$, we obtain the same solution.

Given finite sample data, a common statistical approach is to approximate the expectations with the finite-sample equivalents:

(5) $$0=\frac{1}{N}\sum_{i=1}^{n}\sum_{j\in C_{i}}^{\;}\left[\begin{array}{c} 1\\ X_{i} \end{array}\right]\left(Y_{\mathrm{ijk}}-s_{j}\exp\left({\beta '}_{0k}+X_{i}\beta_{1k}\right)\right),$$

where n is the number of subjects, N is the total number of cells. $C_{i}$ is the set of cells j in subject i. The method of moments commonly uses a finite-sample approximation, known to have an O(1/√N) error (Vaart 2012).

Now, we show that the solution for Equation (5) can be obtained by solving a specific form of Poisson GLM. As $X_{i}$ only depends on i, rearranging Equation (5) gives

(6) $$0=\sum_{i=1}^{n}\left[\begin{array}{c} 1\\ X_{i} \end{array}\right]\left(\sum_{j\in C_{i}}^{\;}Y_{\mathrm{ijk}}-\exp\left({\beta '}_{0k}+X_{i}\beta_{1k}\right)\;\cdot \sum_{j\in C_{i}}^{\;}s_{j}\right).$$

Let$Y_{ik}=\sum_{j\in C_{i}}^{\;}Y_{\mathrm{ijk}}$ and $s_{i}=\;\sum_{j\in C_{i}}^{\;}s_{j}$. Then, the previous equation becomes

(7) $$0=\sum_{i=1}^{n}\left[\begin{array}{c} 1\\ X_{i} \end{array}\right]\left(Y_{ik}-s_{i}\exp\left({\beta '}_{0k}+X_{i}\beta_{1k}\right)\right)=\sum_{i=1}^{n}\left[\begin{array}{c} 1\\ X_{i} \end{array}\right]\left(Y_{ik}-\exp\left(\log s_{i}+{\beta '}_{0k}+X_{i}\beta_{1k}\right)\right),$$

which is exactly the derivative of Poisson GLM log-likelihood with the following regression equation (in R-like syntax).

(8) $$Y_{ik}\;∼\log s_{i}+{\beta '}_{0k}+X_{i}\beta_{1k}.$$

Hence, we converted regression (1), in which the cells indexed by j are the data points, into regression (8), where the subjects indexed by i are the data points. The coefficient of $X_{i}$ is $\beta_{1k}$ for both regressions, so they estimate the same parameter. Note that $\beta_{1k}$ is the logFC of transcript k. While Equation (1) includes $s_{j}$ as an offset for normalization, $s_{i}=\;\sum_{j\in C_{i}}^{\;}s_{j}$ is the offset in Equation (8) for each subject. We call Equation (8) offset-pseudobulk because it aggregates the counts to form a pseudobulk and runs a GLM with an offset variable $s_{i}$.

We emphasize that our argument is stronger than simply saying two different estimators asymptotically converge to the same value. The solution for regression (8) is analytically equivalent to the solution for Equation (5) exactly. Thus, the data-driven estimator ($\hat{\beta_{1k}}$) remains the same. This means that the variance of the estimator ($\mathrm{Var}(\hat{\beta_{1k}})$) is also the same. (You can imagine a random generative procedure to generate multiple datasets. If two approaches give the same $\hat{\beta_{1k}}$ for each dataset, their variances are the same.) This implies that the statistical power and type 1 error rate are also nearly identical between GLMM and offset-pseudobulk.

There can be two sources of discrepancy, though. First, there can be a small difference in order O(1/√N) when we employed finite-sample approximation in Equation (5). The error is negligible in practice as the number of cells is large. Second, there can be an implementation difference in estimating the variance of the estimator ($\hat{\mathrm{Var}}\;(\hat{\beta_{1k}})$) between GLMM and offset-pseudobulk implementations.

### 2.3 Simulations

We used muscat (Crowell *et al.* 2020) to simulate synthetic datasets of varying sizes. To compare different normalization schemes and size factors, we changed the number of subjects (10, 20, 30, 40, 50), the number of cells per subject (100, 200, 500), and the average fold-change (2, 2.5, 3, 3.5, 4). To estimate the runtime of different methods in large data, we change the number of subjects (60, 80, 100) and the number of cells per subject (2000, 4000, 8000). The maximum number of cells was 800 000.

### 2.4 Datasets and software

One1k dataset (Yazar *et al.* 2022) of 982 peripheral blood donors was used to conduct the simulation. We selected the top 5 most common cell types and subjects with more than 50 cells for each type which left 403 subjects. Next, we selected 10, 20, 40, 80, and 120 subjects and divided them equally into two groups randomly to form cases and controls. Finally, the expression of all genes above a mean threshold of 0.1 was compared for each cell type. The number of cells per donor was set to 10. To simulate the imbalance of the number of cells per subject, we injected random noise with variance 0.25 * average number of cells per donor.

We repeated the above step 100 times to quantify the true variability of estimates. For each trial, we saved the *P*-values of the two methods (offset-pseudobulk and NB GLMM) and plotted the observed quantile against the theoretical quantile to assess the validity.

We repeated the previous step with transcripts with a mean below 0.1 to evaluate the numerical stability. We counted the number of excluded or non-convergent tests produced by glmGamPoi and NEBULA.

To measure the runtime, we used all 982 donors. The CD4 helper T cells, the most common cell type, were used for the benchmark. We varied the number of cells per donor from 20 to 80 and the number of subjects from 10 to 200. We repeated the process 20 times, assuming that a typical scRNA-seq dataset contains 20 different cell types. The number of tested transcripts was set to 10 000. The benchmark was conducted on an Intel Xeon Silver 4116 CPU. The number of threads was restricted to 2. The version of glmGamPoi was 1.12.2, and NEBULA is 1.5.0 on R 4.3.1. Note that the current version of glmGamPoi only runs in a single thread.

In all analyses, we executed glmGamPoi with the default option using the “glm_gp” command. We used the “pseudobulk” command in glmGamPoi to create the aggregated counts. “glm_gp”’s default offset is the column sum of the count matrix divided by a constant. We supplied the sum of all transcript counts as a size factor for NEBULA through the “offset” option. This was computed by the “colMeans” function applied to the count matrix.

## 3 Results

### 3.1 Offset-pseudobulk produces the same result as GLMMs

The logFC estimates of offset-pseudobulk and NB GLMM were nearly identical across all 5 cell types and 100 trials. Figure 1 shows the estimates from 40 subjects in memory CD4 T cells. The result from one of the trials is shown in Fig. 1a. The variance of the estimate from 100 repetitions was also highly concordant (Fig. 1b), which means that the estimate’s variability due to random sampling was also equal in both methods. The two observations confirm our finding that the logFC estimate $\hat{\beta_{1k}}$ and its variance $\mathrm{Var}(\hat{\beta_{1k}})\;$are identical in both methods (see Supplementary Figs S1–S25 for other cell types).

**Figure 1.**
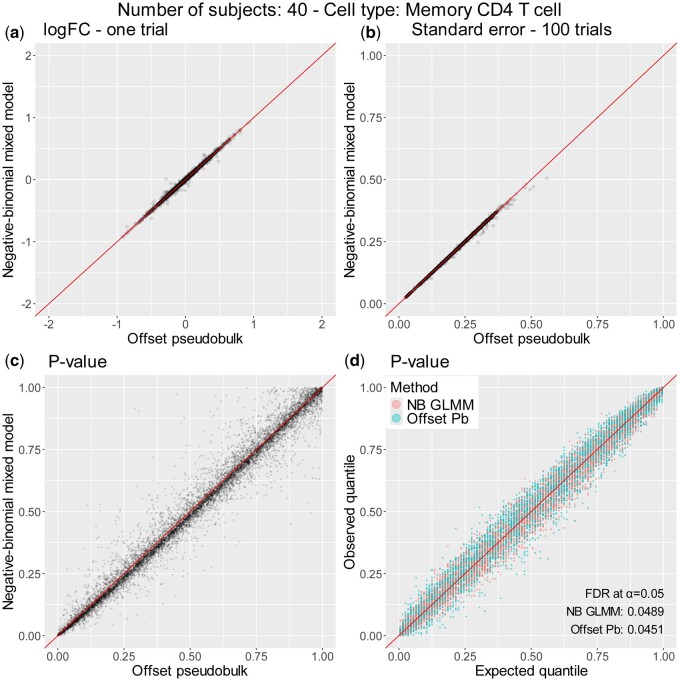
Comparison of offset-pseudobulk and negative-binomial GLMM. Total 40 subjects and 10 cells per donor. (a) Point estimates of the two methods in one of the 100 trials. (b) Standard error of two methods computed from 100 trials. (c) *P-*values of the two methods in 100 trials across transcripts with a mean above 0.1. (d) *P-*values of the two methods plotted against the expected distribution across 100 trials.

In practice, only $\hat{\beta_{1k}}$ is directly available, so $\mathrm{Var}(\hat{\beta_{1k}}$) is estimated from data to obtain $\hat{\mathrm{Var}}(\hat{\beta_{1k}})$. The variance estimate $\hat{\mathrm{Var}}(\hat{\beta_{1k}})\;$may vary across method implementations, although the true variance $\mathrm{Var}(\hat{\beta_{1k}})\;$is the same. *P*-value, which is computed from $\hat{\mathrm{Var}}\left(\hat{\beta_{1k}}\right)$, of the methods are therefore less identical than their logFC point estimates $\hat{\beta_{1k}}$ (Fig. 1c). We find that glmGamPoi generally produces slightly conservative (less powerful and fewer false positives) *P-*values compared to NEBULA when the number of subjects is small (≤20, Supplementary Figs S1–S10). For larger samples (≥40), the gap between the methods narrows (Fig. 1c and Supplementary Figs S11–S25). However, looking at the low threshold regions (α = 0.01, 0.05, and 0.1), the gap that leads to pseudobulk’s slightly lower FDRs under the null remains (Supplementary Figs S26–S30).

We found that the *P-*values of both methods were well-calibrated when compared to the expected distribution (which is uniform) across ranges of conditions (Fig. 1d and Supplementary Figs S1–S25).

Finally, we ran non-null simulations with varying levels of average logFC between conditions (see Section 2). As in previous results, the point estimates between glmGamPoi and NEBULA were highly concordant (see Supplementary Figs S31–S35). As discussed in detail in the Section 4, the concordance is not specific to glmGamPoi. It applies to other GLM-based software, such as DESeq2 and edgeR, with different normalization schemes (Supplementary Figs S36–S40).

### 3.2 Scalability and stability of offset-pseudobulk compared to GLMM

**Figure 2.**
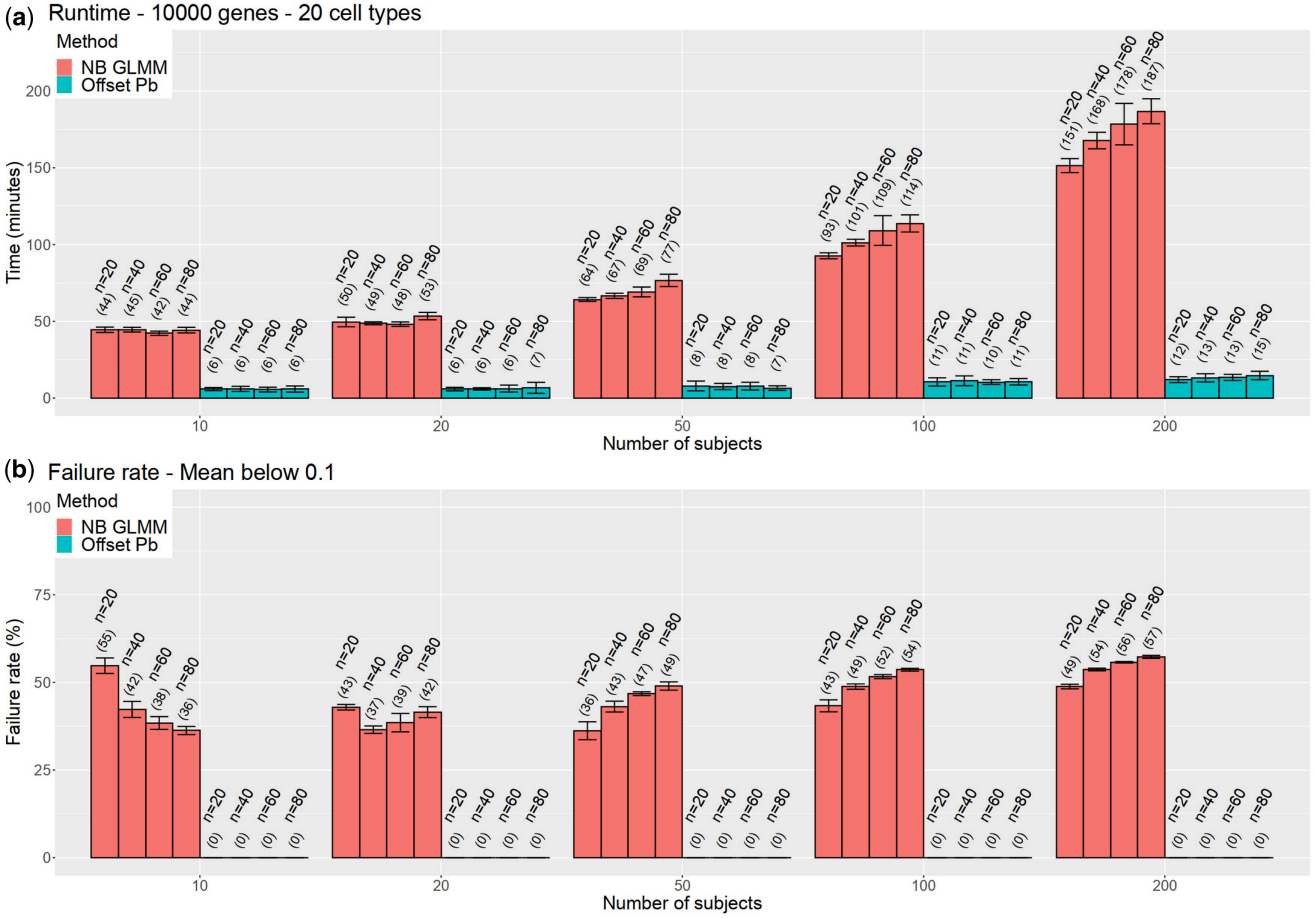
Runtime and stability of offset-pseudobulk and negative-binomial GLMM. (a) The runtime of the two methods in 10 000 transcripts and 20 cell types. (b) The failure rate of the two methods in transcripts with a mean below 0.1.

Despite these striking similarities between the two approaches, offset-pseudobulk was faster and more stable than NB GLMM. The size of the aggregated counts only depends on the number of subjects, so the burden of fitting GLM in pseudobulk does not depend on the number of cells. The aggregation step scales linearly with the increasing number of cells, but summing rows and columns of a sparse matrix is very efficient. Hence, the method is suitable for large data. In contrast, the runtime of NB GLMM depends on both numbers because it uses a cell-level count matrix as an input. Thus, the speed gap of pseudobulk and GLMM widens as the data gets larger. Figure 2a shows that at *N* = 200 and 80 cells per subject, running DGE for 20 cell types (10K genes) takes only 15 minutes in pseudobulk but 3 hours in NB GLMM. In the future, as the data size grows, this difference will become even more prominent. We confirmed the prediction using simulated data from muscat where the gap widens up to 600 times (Supplementary Fig. S41).

Also, offset pseudobulk is more robust to low-expression transcripts. In scRNA-seq, the majority of transcripts exhibit low counts. Offset pseudobulk produces correct estimates in such a case because it fits a GLM with a convex loss function. On the contrary, NB GLMM frequently drops low-expression transcripts or fails to converge. We show this in Fig. 2b, where we plotted the failure rate. For transcripts with a mean below 0.1 (but above 0), GLMM fails in 30%–60% of the transcripts.

## 4 Discussion

We proposed offset-pseudobulk as a convenient replacement for GLMMs in case-control DGE analysis of scRNA-seq data. The approach is substantially faster and produces nearly identical results to negative-binomial GLMM. Furthermore, it is easy to use in practice because it only requires tweaking the options of existing packages. For example, running glmGamPoi on sum-aggregated counts will return point estimates nearly identical to negative-binomial GLMM if we properly use the size factor as an offset per cell.

Offset-pseudobulk is faster, more convenient, and numerically stable when the expression level is low. Although glmGamPoi tends to produce slightly conservative *P*-values compared to NEBULA in a small sample, the problem disappears in large samples. Hence, if one tries to perform case-control DGE in large samples, we recommend using offset-pseudobulk instead of GLMM.

The paper’s suggestion is compatible with any interpretations of the offset variable. In bulk sequencing studies, offsets are normalization factors that correct library size differences across samples. In single-cell studies, size factors additionally correct a cell’s overall transcript abundance. For example, two cells with otherwise similar biological characteristics may have different expression levels solely due to their overall size. We do not impose any of these specific interpretations in our argument, allowing the theory to be applied to any offset.

To demonstrate this point, we show that one can always find a way to reach the same result as a pseudobulk with an arbitrary offset using a mixed model. By assigning the subject’s offset (used in pseudobulk normalization) divided by the subject’s cell number equally to its cells, mixed models produce identical results. We confirm this in DESeq2 and edgeR, the two popular software for pseudobulk DGE analysis (Supplementary Figs S36–S40). Therefore, any pseudobulk can be reproduced using a mixed model and vice versa.

The theory presented in the paper is applicable when all cells from the same subject belong to the same comparison group, which is often the case in case-control comparisons. This assumption is the key idea of converting Equation (5) to (6) in the Section 2. We can only guarantee that the two approaches will coincide when the assumption is met. The equivalence of mixed models and pseudobulk methods breaks down when this condition is violated. For instance, this occurs when one compares different cell types from the same group of subjects.

Another problem with comparing cell types from the same group of subjects is that cell types are assigned adaptively after looking at the data through unsupervised learning, such as the Louvain/Leiden algorithm. The theory handling this situation is mathematically sophisticated and is studied in the selective inference literature, which is beyond the scope of this paper (Neufeld *et al.* 2023, Gao *et al.* 2024).

The work warrants future developments. One could develop a robust variance estimator of the logFC that does not produce conservative *P-*values in small samples so that offset-pseudobulk can be reliably applied to smaller data. Further computational speed-ups will be possible through multithreading, which is not supported by current software. Finally, we caution the reader that our finding only applies when the condition of the cells is completely determined at the subject level (i.e. case-control design). When the condition varies within a subject (as in CRISPR perturbation studies), mixed models and pseudobulk may produce drastically different results.

## Supplementary Material

btae498_Supplementary_Data

## Data Availability

An example code for implementing offset pseudobulk can be found at https://github.com/hanbin973/pseudobulk_is_mm. The codes that produce the results of the paper are also stored in the same repository.
