# Supplementary material for: Pseudobulk with proper offsets has the same statistical properties as generalized linear mixed models in single-cell case-control studies
Source: Bioinformatics. 2024 Aug 8;40(8):btae498. doi: 10.1093/bioinformatics/btae498 (PMC11343365; doi:10.1093/bioinformatics/btae498)
Supplement: btae498_Supplementary_Data [file btae498_supplementary_data.pdf]

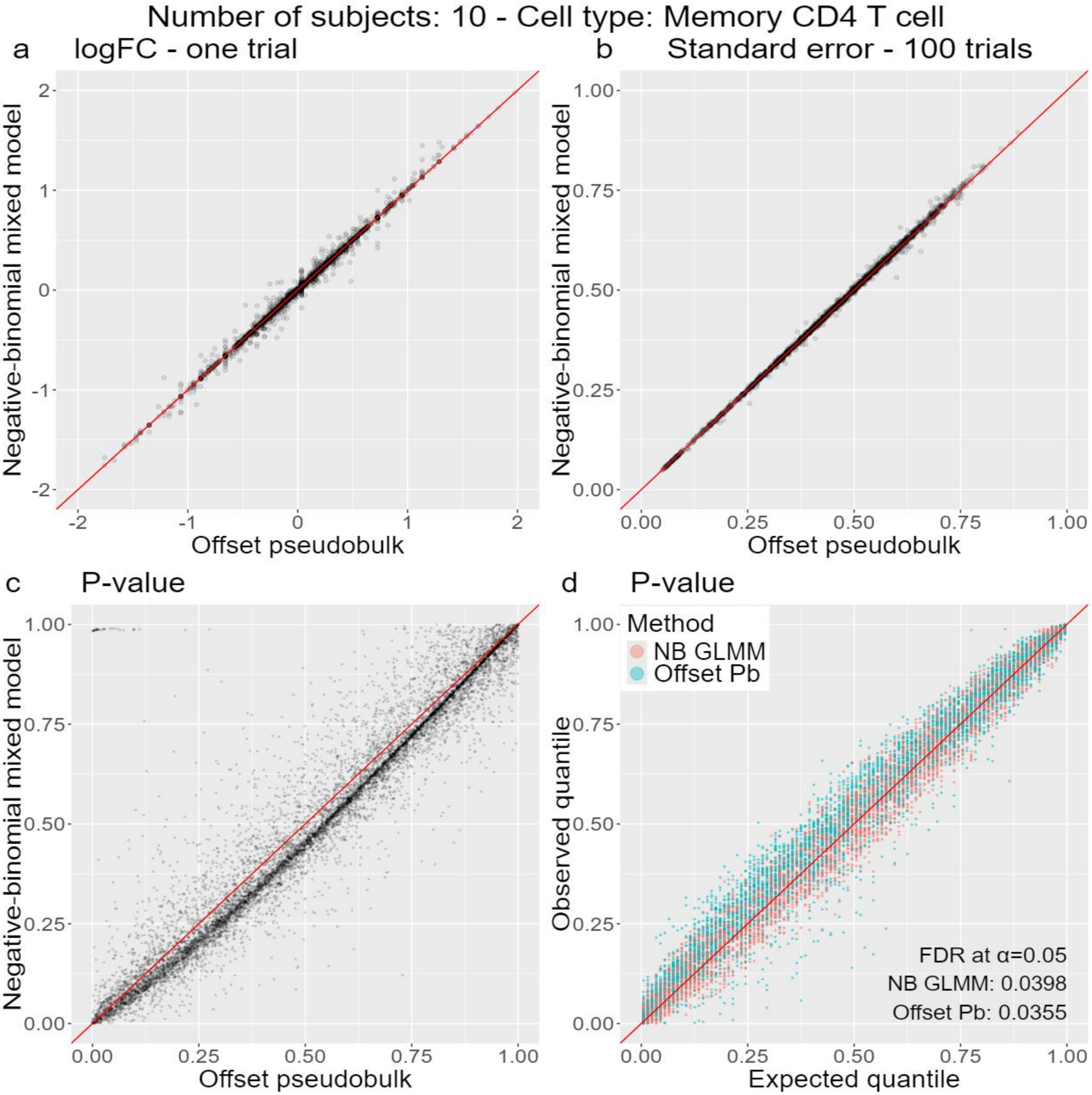

Supplementary Figure 1 a. Point estimates of the two methods in one of the 100 trials. **b.** Standard error of two methods computed from 100 trials. **c.** *P*-values of the two methods in 100 trials across transcripts with a mean above 0.1. **d.** *P*-values of the two methods plotted against the expected distribution across 100 trials.

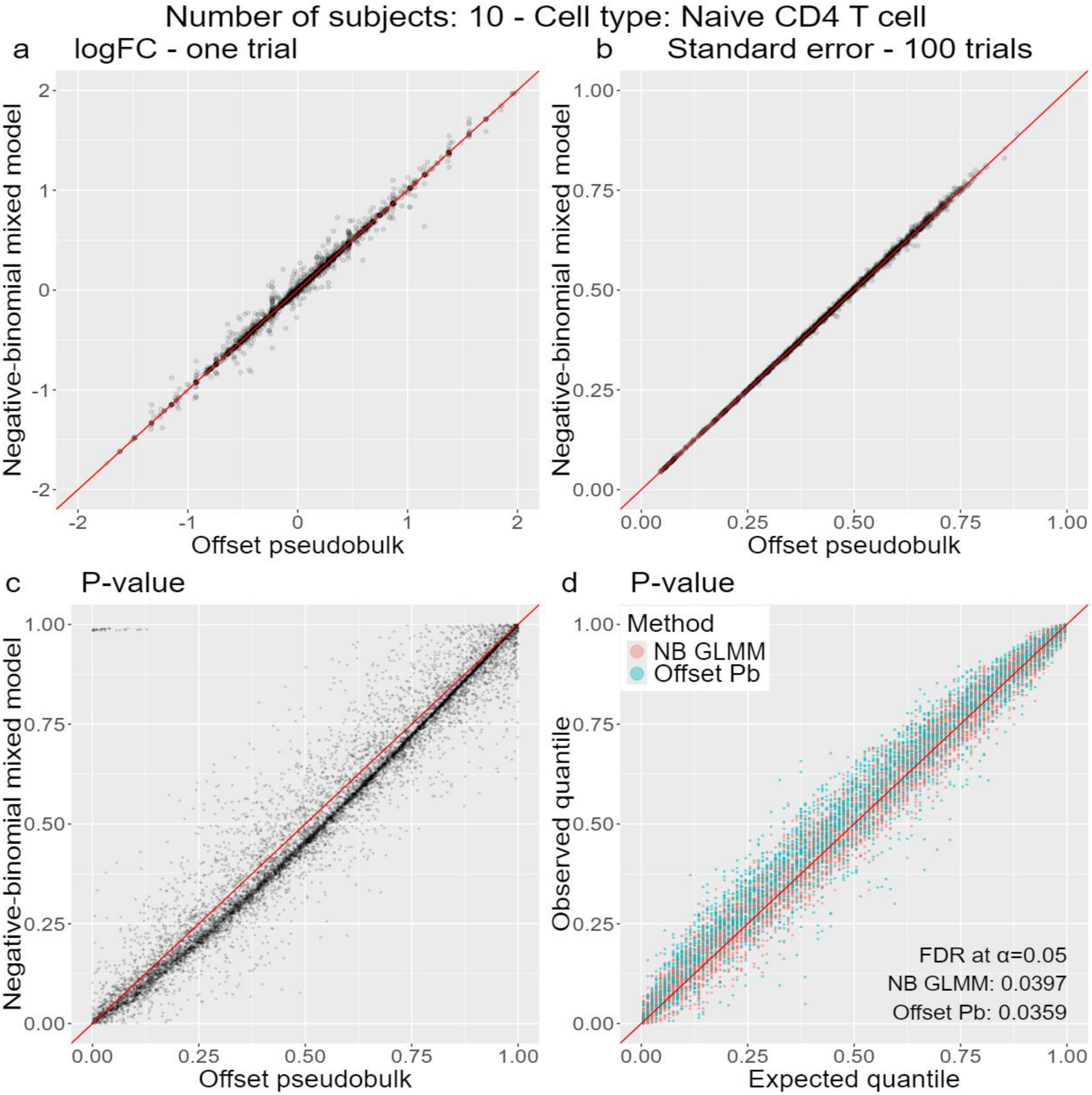

Supplementary Figure 2 a. Point estimates of the two methods in one of the 100 trials. **b.** Standard error of two methods computed from 100 trials. **c.** *P*-values of the two methods in 100 trials across transcripts with a mean above 0.1. **d.** *P*-values of the two methods plotted against the expected distribution across 100 trials.

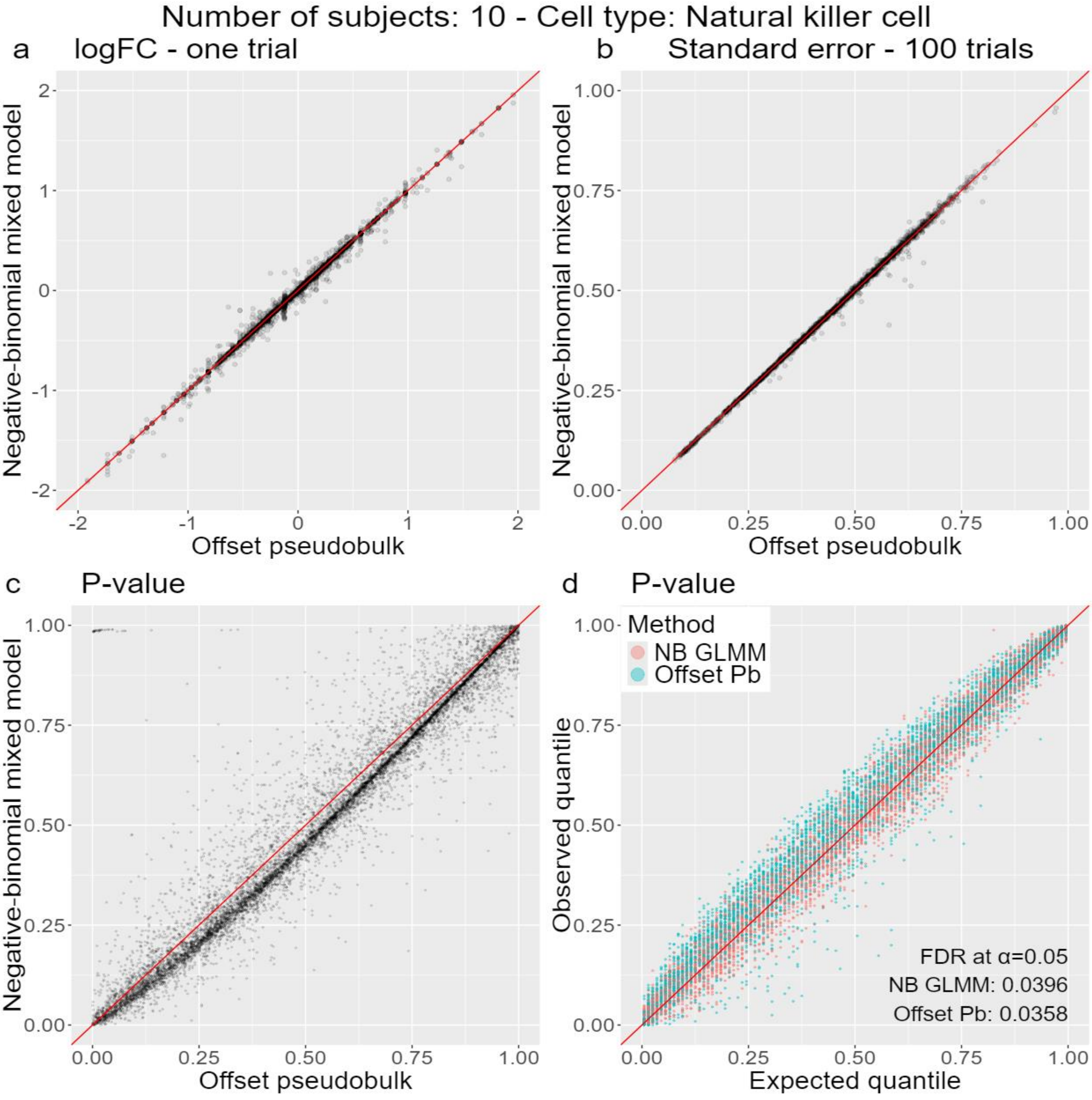

Supplementary Figure 3 a. Point estimates of the two methods in one of the 100 trials. **b.** Standard error of two methods computed from 100 trials. **c.** *P*-values of the two methods in 100 trials across transcripts with a mean above 0.1. **d.** *P*-values of the two methods plotted against the expected distribution across 100 trials.

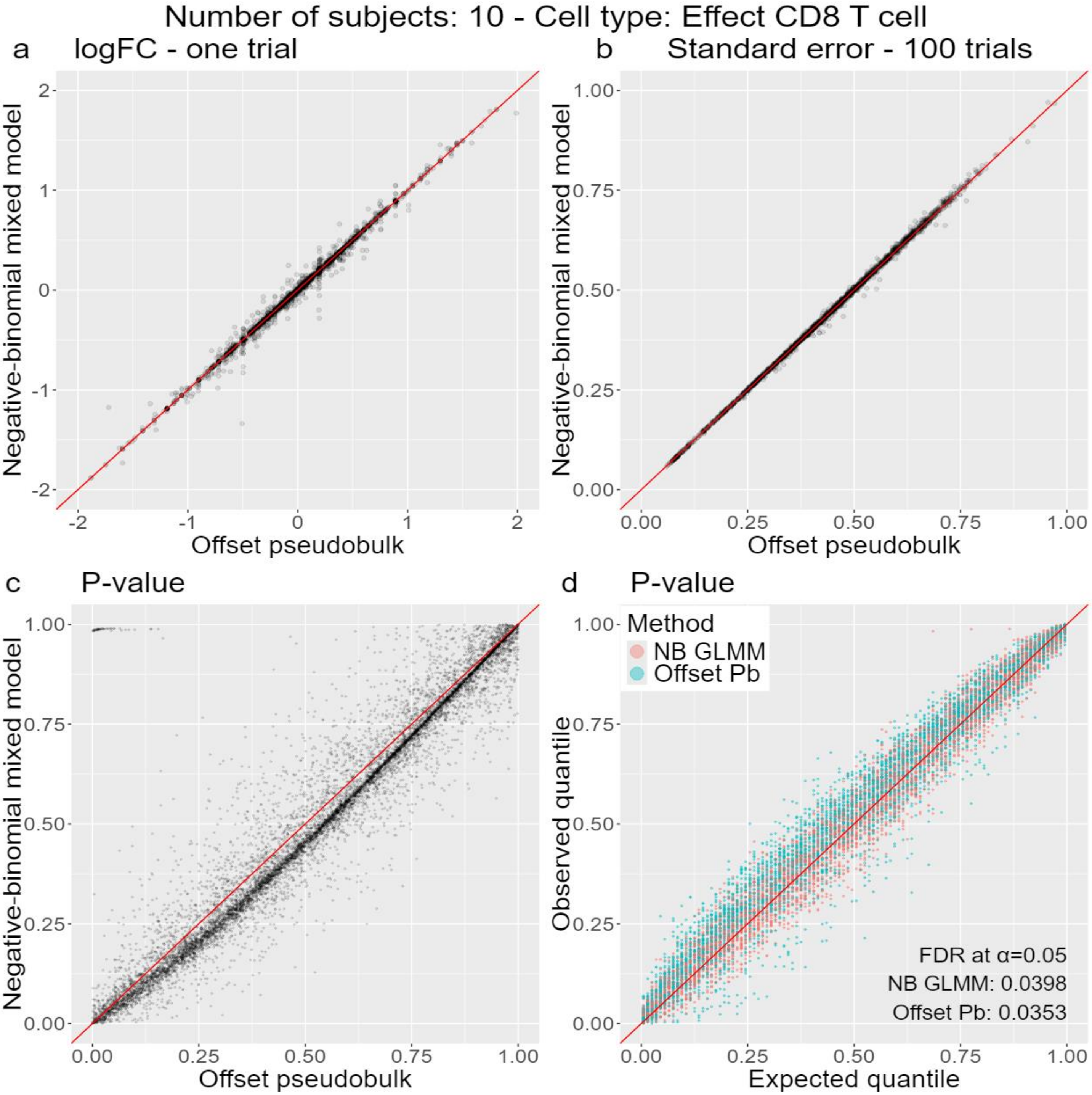

Supplementary Figure 4 a. Point estimates of the two methods in one of the 100 trials. **b.** Standard error of two methods computed from 100 trials. **c.** *P*-values of the two methods in 100 trials across transcripts with a mean above 0.1. **d.** *P*-values of the two methods plotted against the expected distribution across 100 trials.

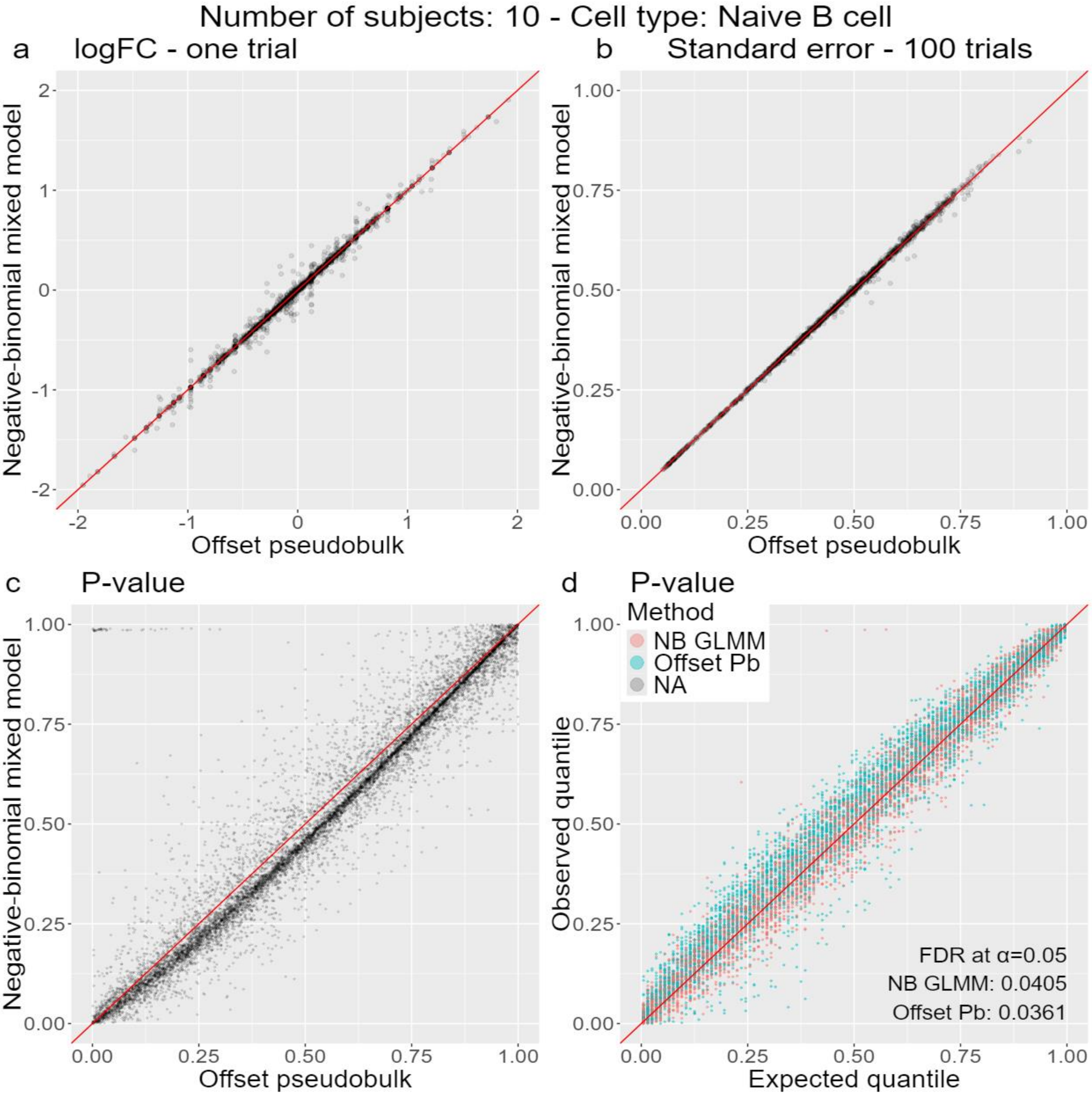

Supplementary Figure 5 a. Point estimates of the two methods in one of the 100 trials. **b.** Standard error of two methods computed from 100 trials. **c.** *P*-values of the two methods in 100 trials across transcripts with a mean above 0.1. **d.** *P*-values of the two methods plotted against the expected distribution across 100 trials.

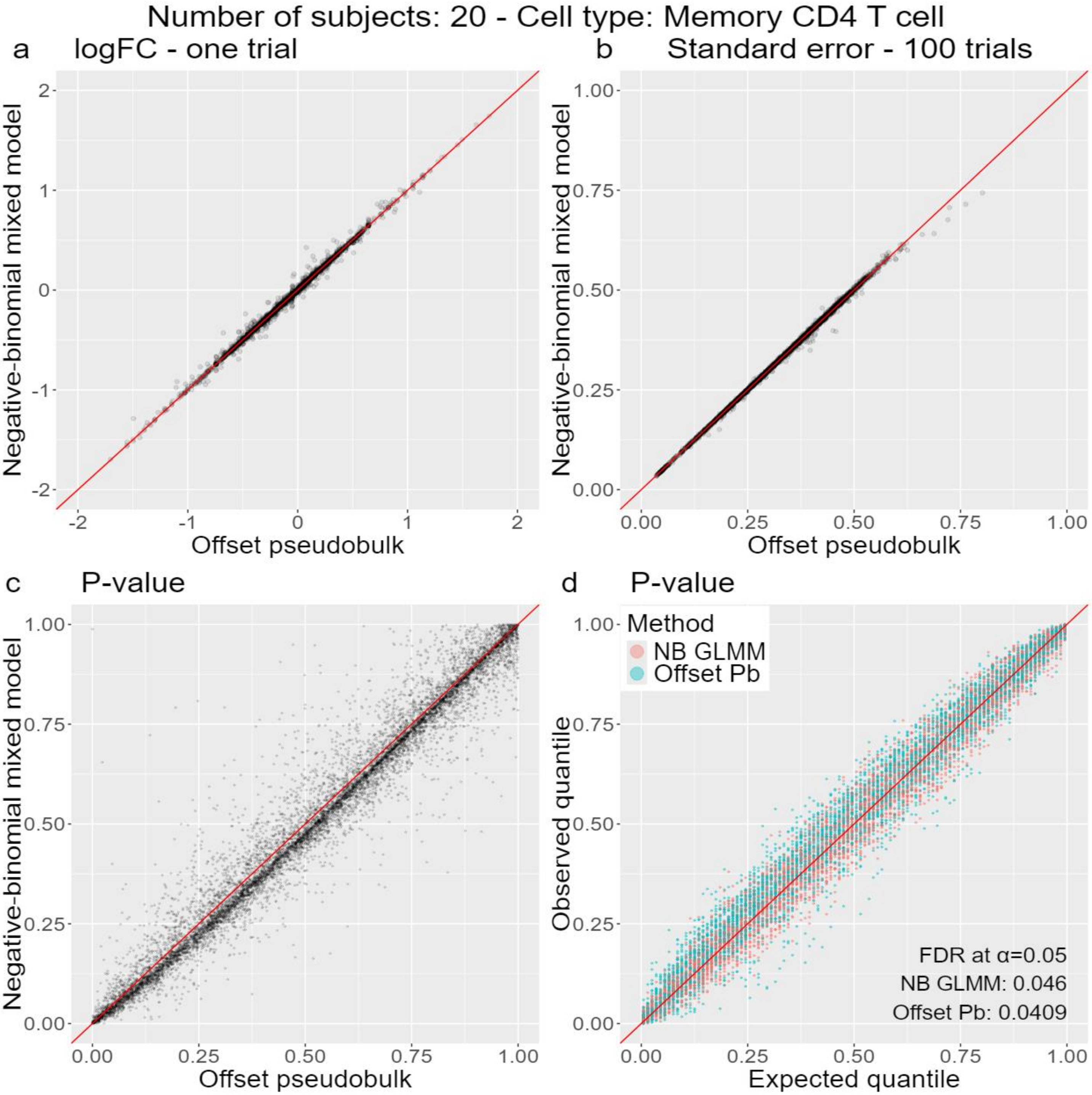

Supplementary Figure 6 a. Point estimates of the two methods in one of the 100 trials. **b.** Standard error of two methods computed from 100 trials. **c.** *P*-values of the two methods in 100 trials across transcripts with a mean above 0.1. **d.** *P*-values of the two methods plotted against the expected distribution across 100 trials.

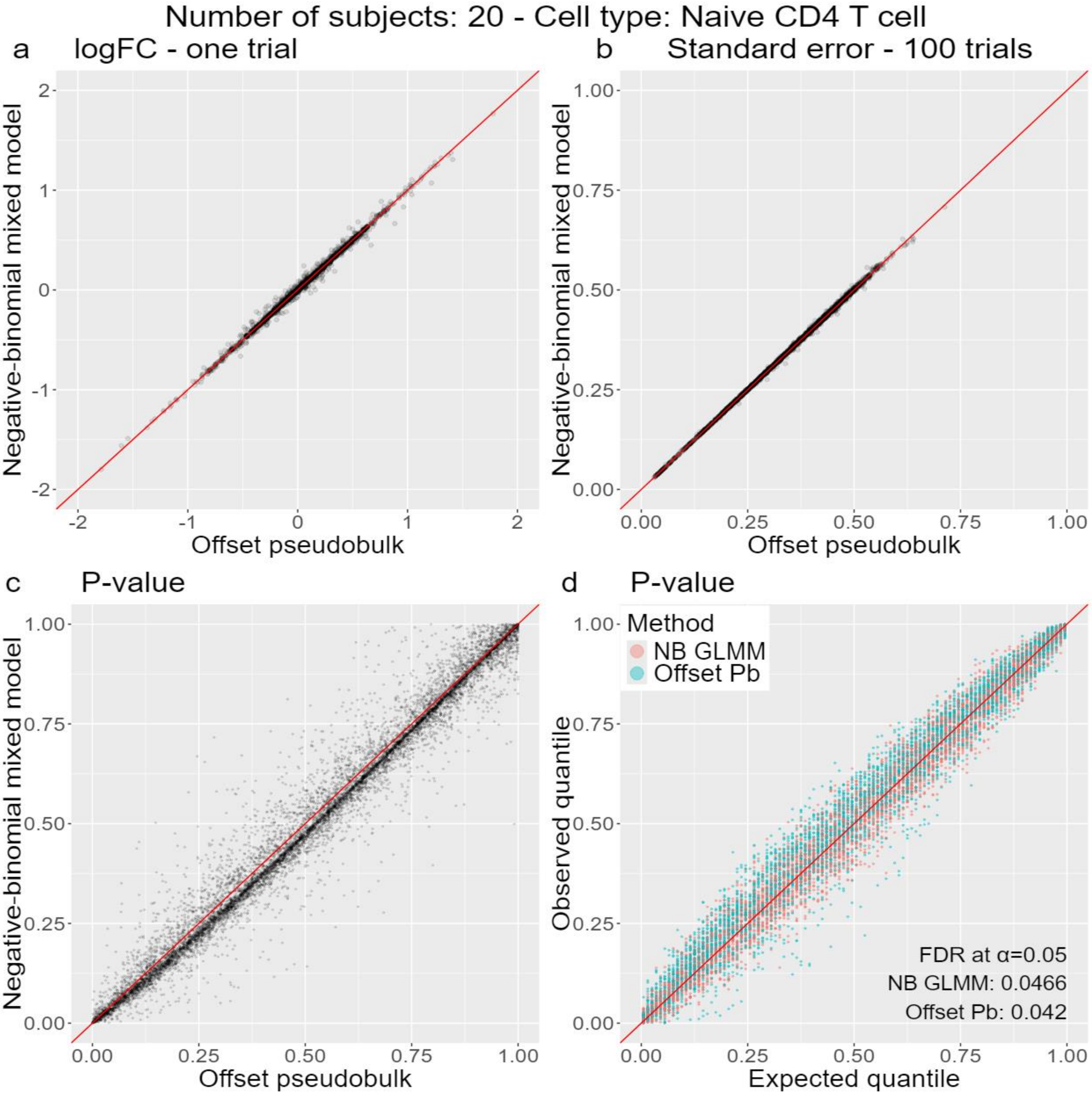

Supplementary Figure 7 a. Point estimates of the two methods in one of the 100 trials. **b.** Standard error of two methods computed from 100 trials. **c.** *P*-values of the two methods in 100 trials across transcripts with a mean above 0.1. **d.** *P*-values of the two methods plotted against the expected distribution across 100 trials.

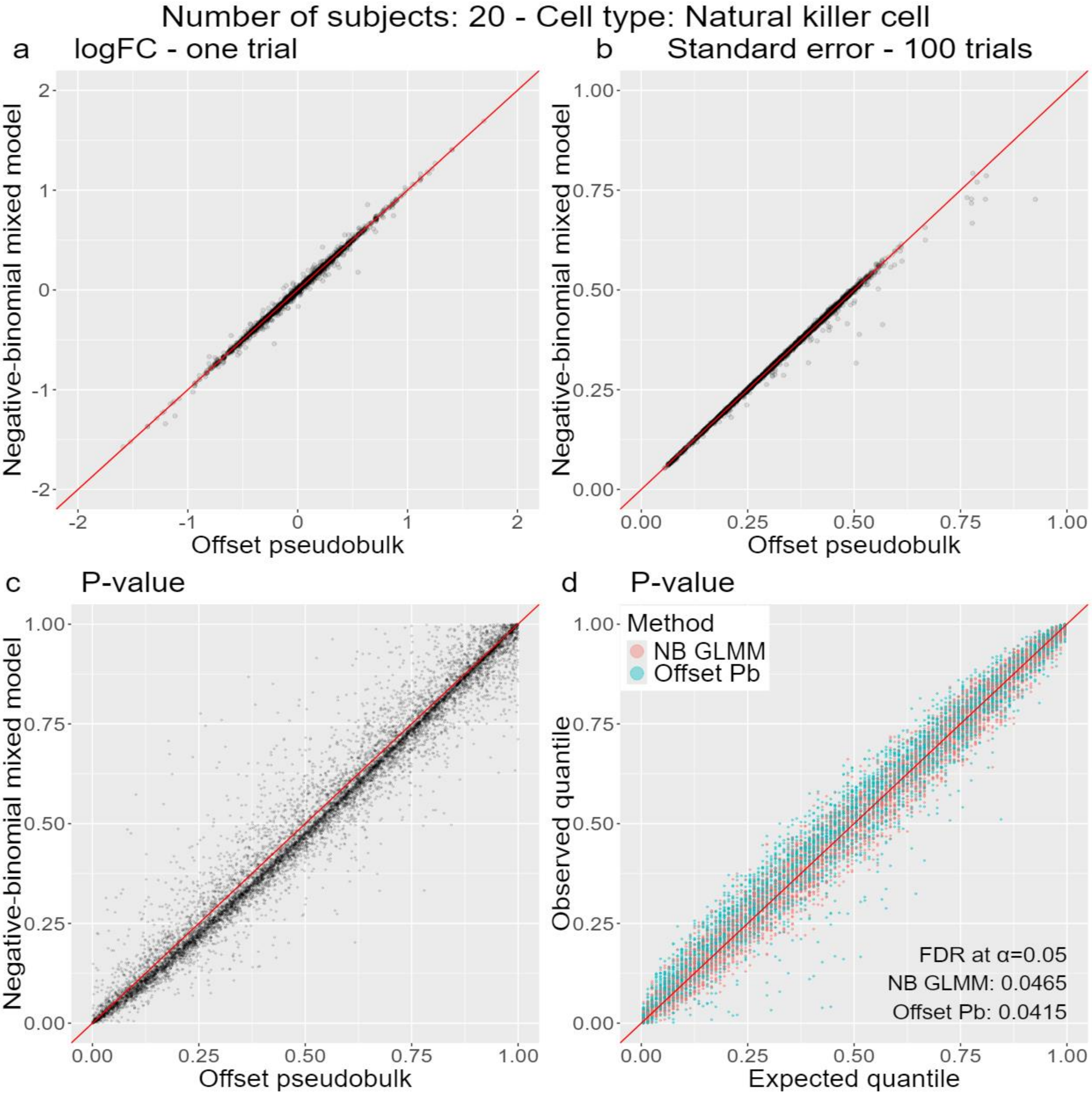

Supplementary Figure 8 a. Point estimates of the two methods in one of the 100 trials. **b.** Standard error of two methods computed from 100 trials. **c.** *P*-values of the two methods in 100 trials across transcripts with a mean above 0.1. **d.** *P*-values of the two methods plotted against the expected distribution across 100 trials.

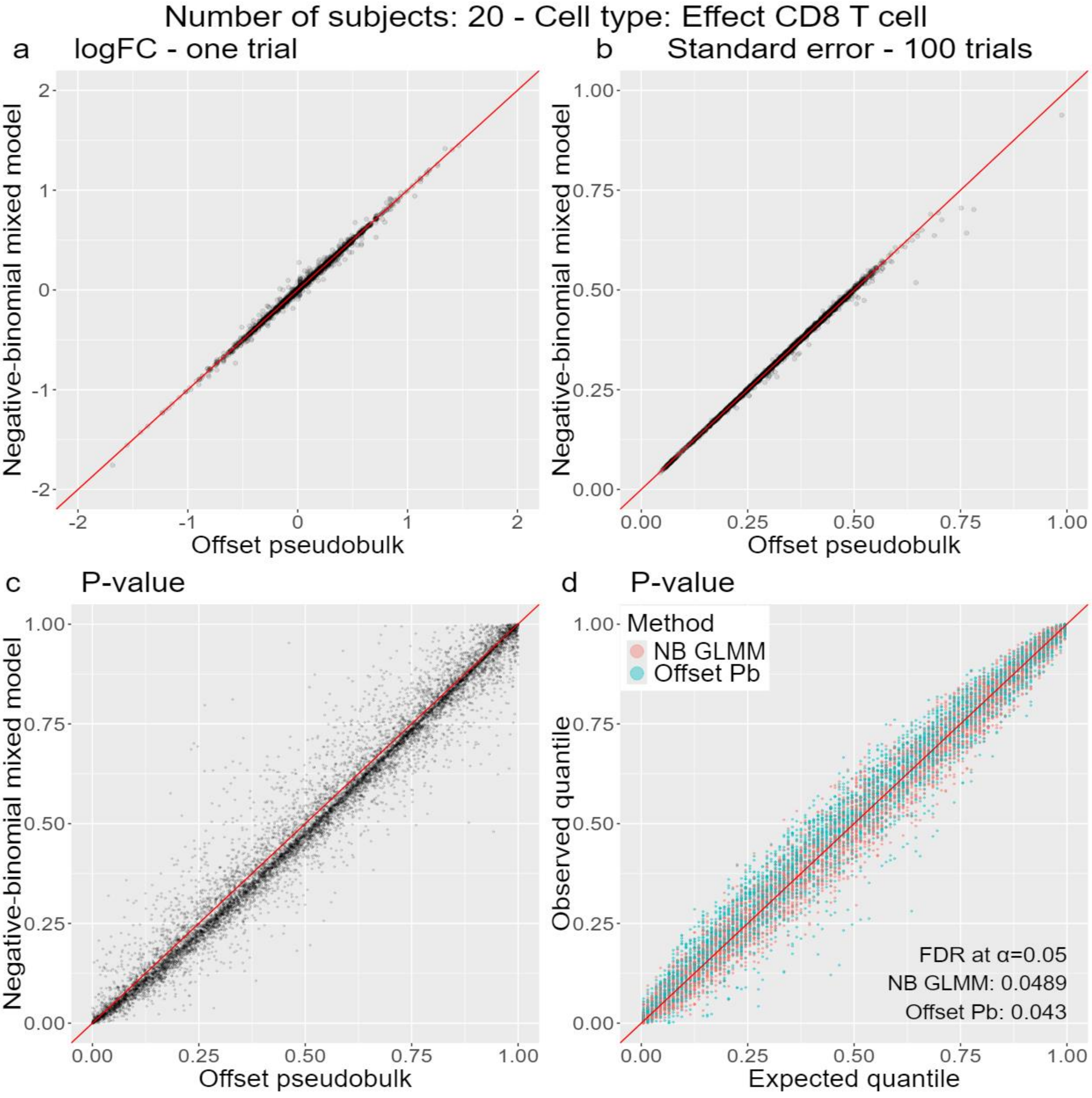

Supplementary Figure 9 a. Point estimates of the two methods in one of the 100 trials. **b.** Standard error of two methods computed from 100 trials. **c.** *P*-values of the two methods in 100 trials across transcripts with a mean above 0.1. **d.** *P*-values of the two methods plotted against the expected distribution across 100 trials.

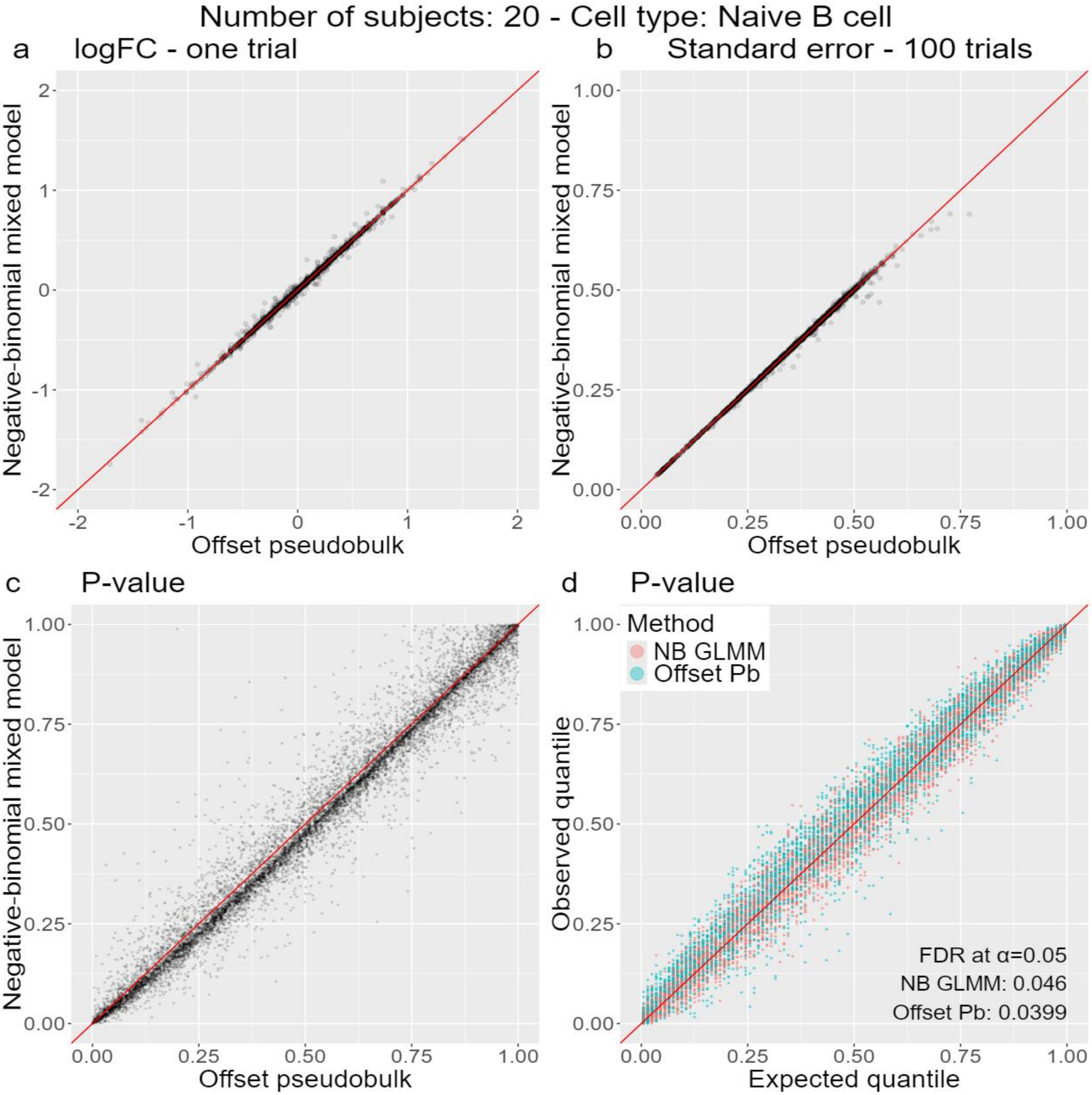

Supplementary Figure 10 a. Point estimates of the two methods in one of the 100 trials. **b.** Standard error of two methods computed from 100 trials. **c.** *P*-values of the two methods in 100 trials across transcripts with a mean above 0.1. **d.** *P*-values of the two methods plotted against the expected distribution across 100 trials.

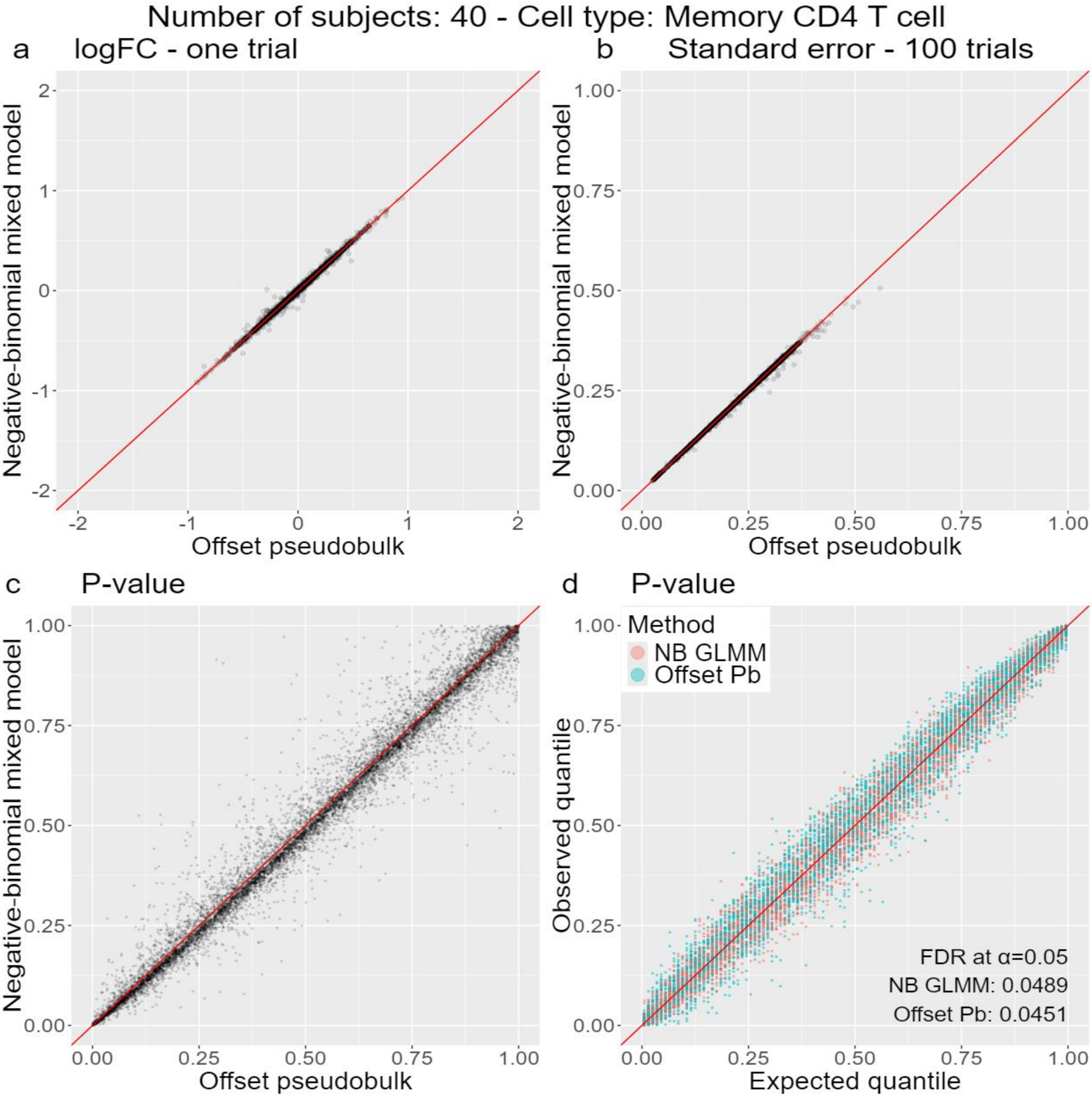

Supplementary Figure 11 a. Point estimates of the two methods in one of the 100 trials. **b.** Standard error of two methods computed from 100 trials. **c.** *P*-values of the two methods in 100 trials across transcripts with a mean above 0.1. **d.** *P*-values of the two methods plotted against the expected distribution across 100 trials.

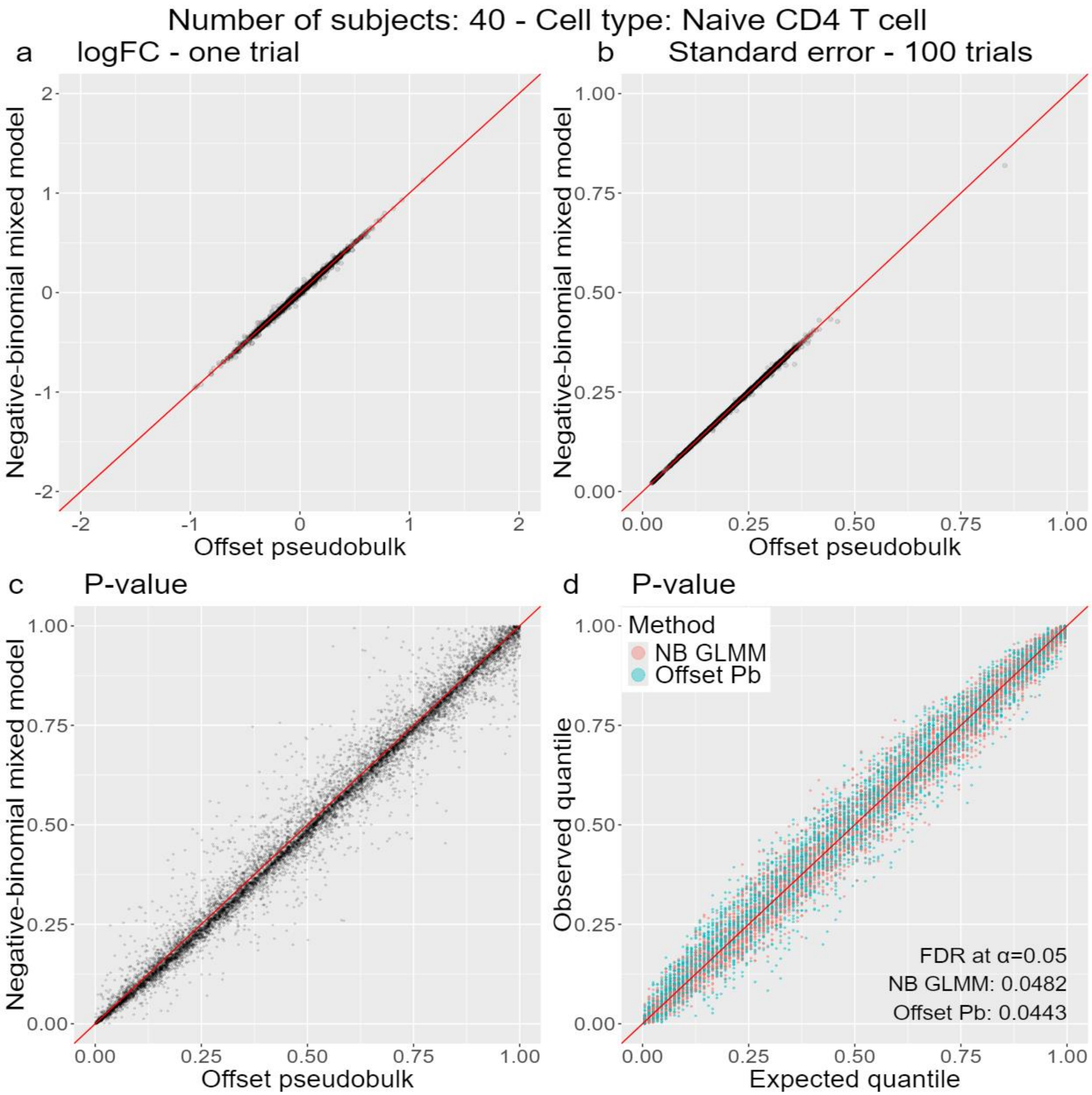

Supplementary Figure 12 a. Point estimates of the two methods in one of the 100 trials. **b.** Standard error of two methods computed from 100 trials. **c.** *P*-values of the two methods in 100 trials across transcripts with a mean above 0.1. **d.** *P*-values of the two methods plotted against the expected distribution across 100 trials.

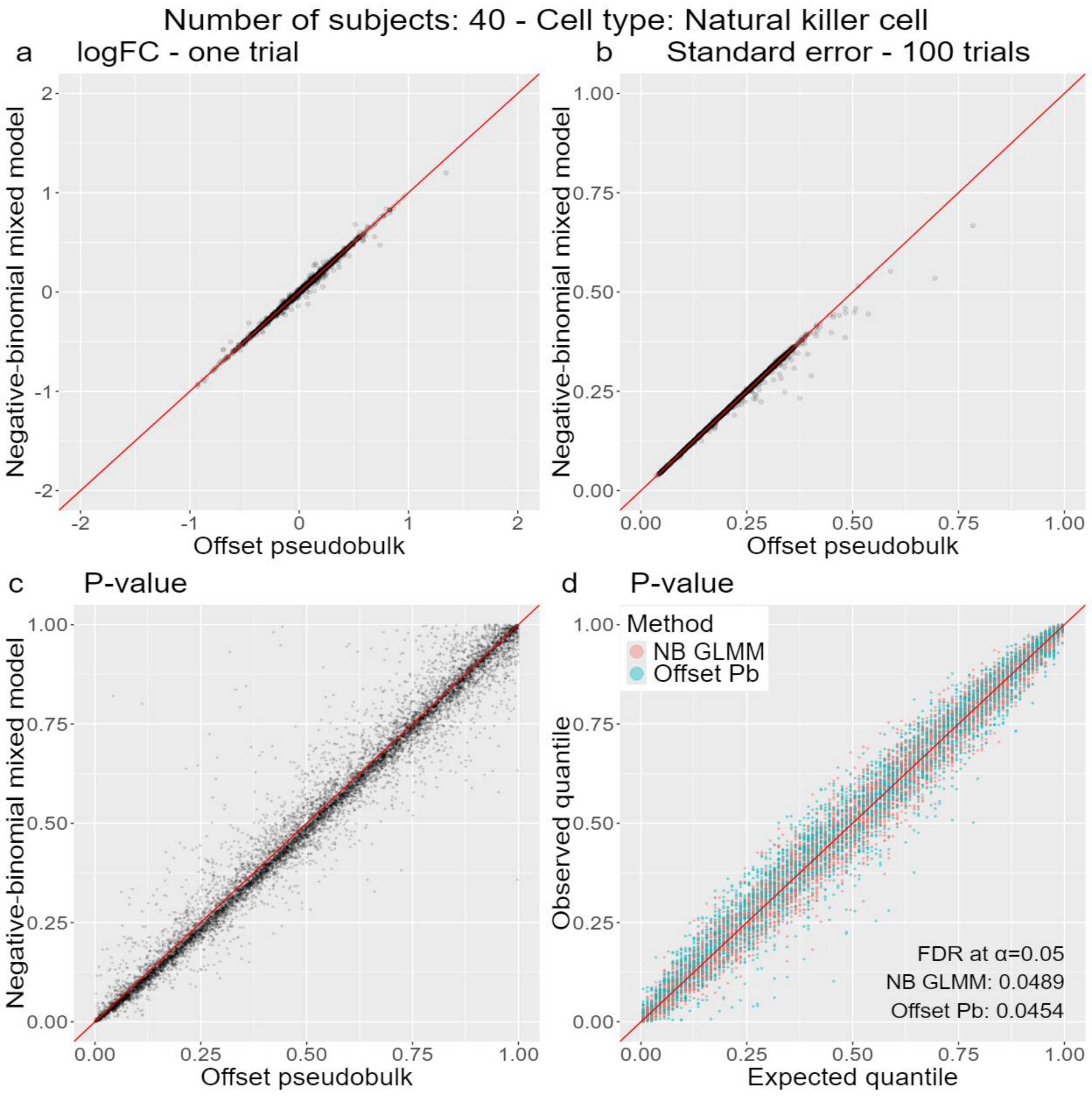

Supplementary Figure 13 a. Point estimates of the two methods in one of the 100 trials. **b.** Standard error of two methods computed from 100 trials. **c.** *P*-values of the two methods in 100 trials across transcripts with a mean above 0.1. **d.** *P*-values of the two methods plotted against the expected distribution across 100 trials.

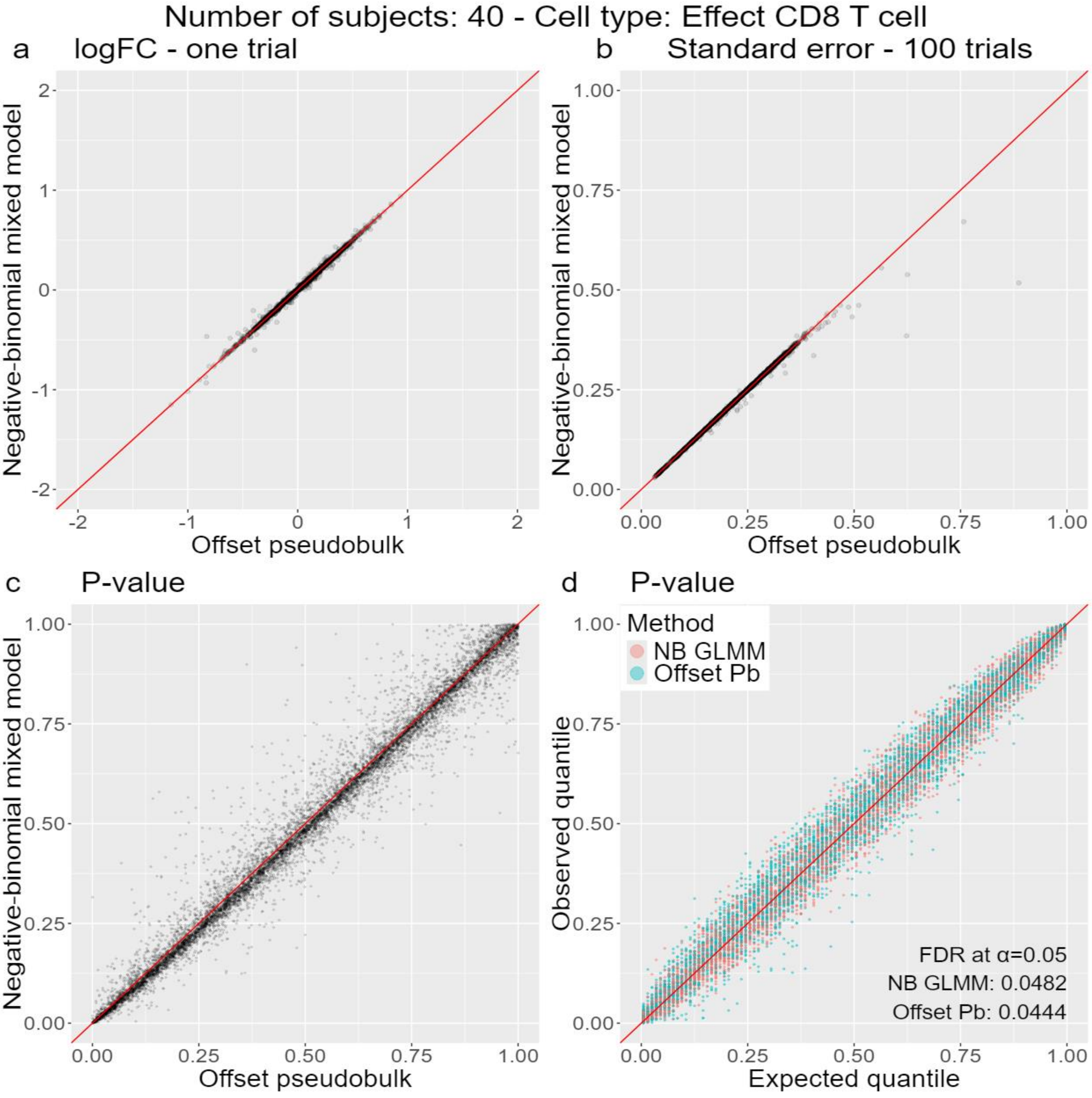

Supplementary Figure 14 a. Point estimates of the two methods in one of the 100 trials. **b.** Standard error of two methods computed from 100 trials. **c.** *P*-values of the two methods in 100 trials across transcripts with a mean above 0.1. **d.** *P*-values of the two methods plotted against the expected distribution across 100 trials.

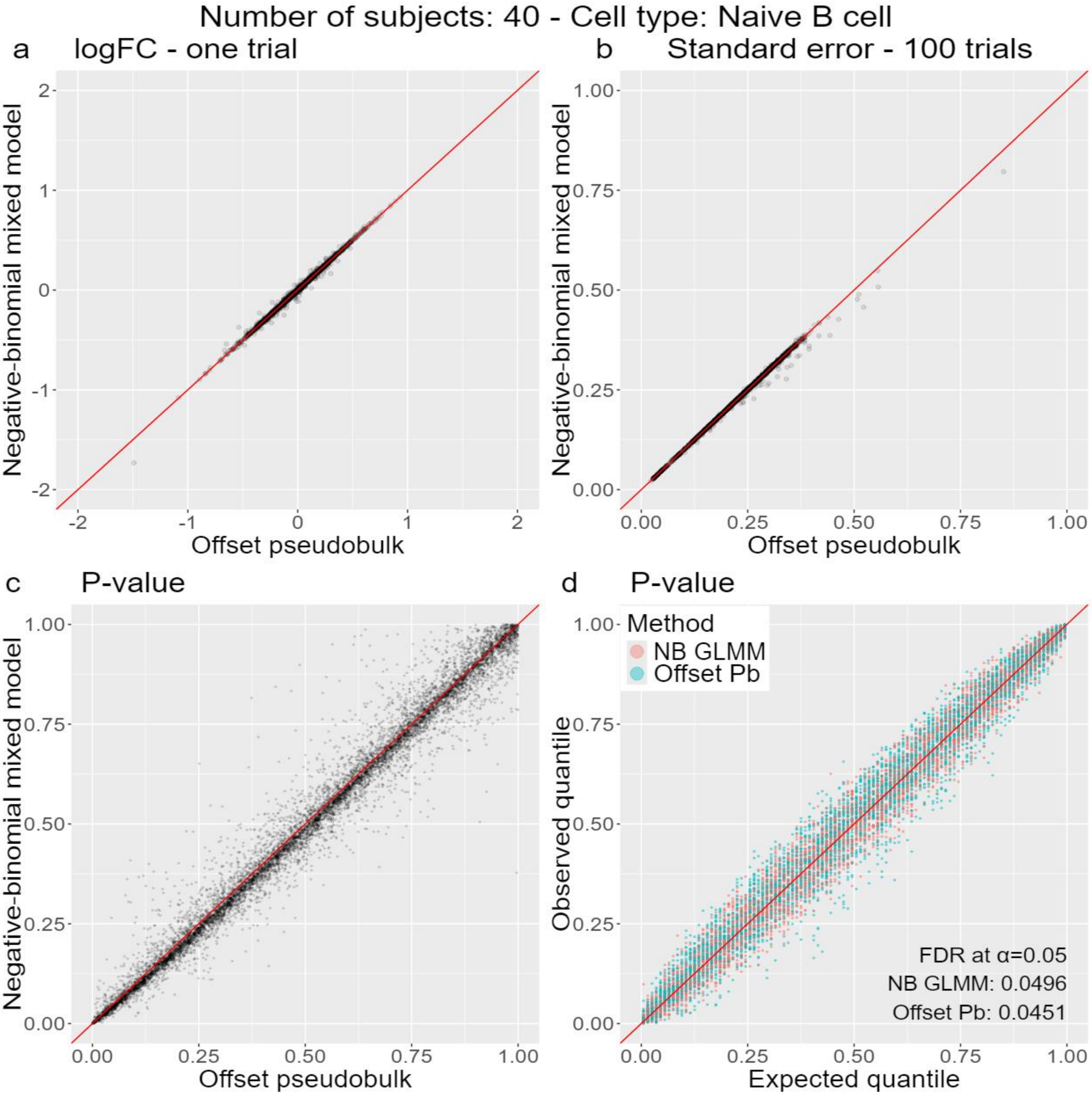

Supplementary Figure 15 a. Point estimates of the two methods in one of the 100 trials. **b.** Standard error of two methods computed from 100 trials. **c.** *P*-values of the two methods in 100 trials across transcripts with a mean above 0.1. **d.** *P*-values of the two methods plotted against the expected distribution across 100 trials.

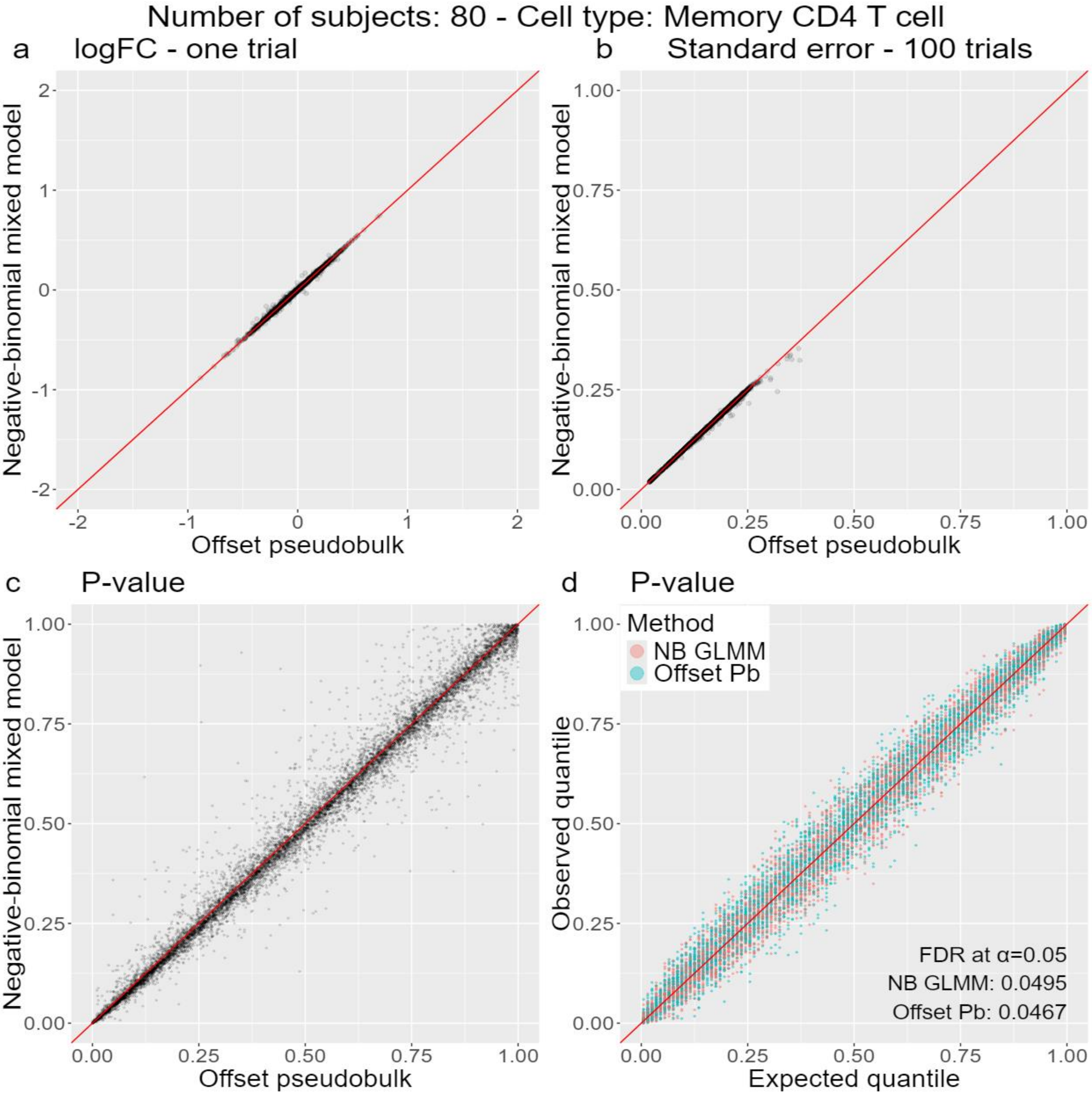

Supplementary Figure 16 a. Point estimates of the two methods in one of the 100 trials. **b.** Standard error of two methods computed from 100 trials. **c.** *P*-values of the two methods in 100 trials across transcripts with a mean above 0.1. **d.** *P*-values of the two methods plotted against the expected distribution across 100 trials.

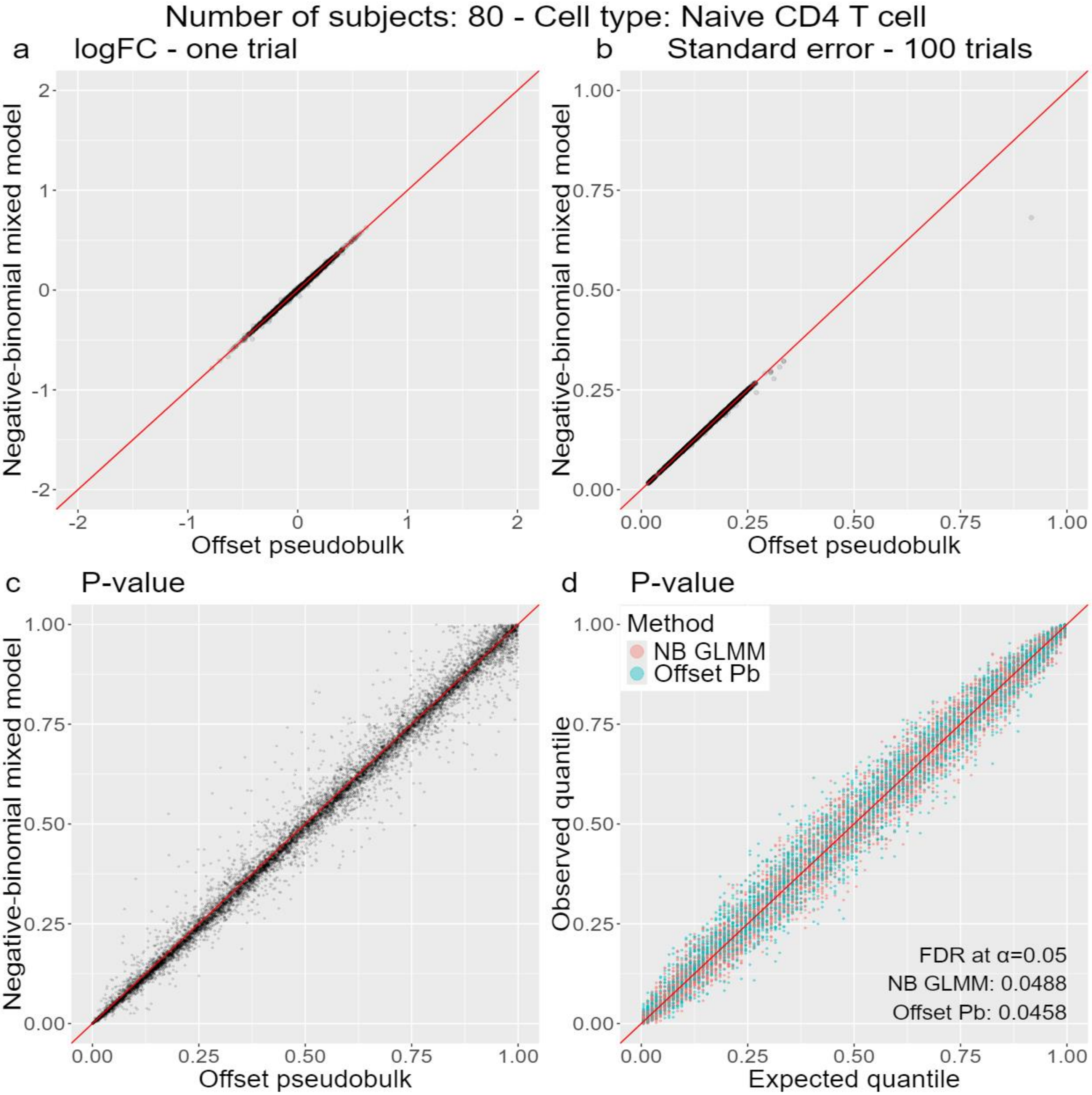

Supplementary Figure 17 a. Point estimates of the two methods in one of the 100 trials. **b.** Standard error of two methods computed from 100 trials. **c.** *P*-values of the two methods in 100 trials across transcripts with a mean above 0.1. **d.** *P*-values of the two methods plotted against the expected distribution across 100 trials.

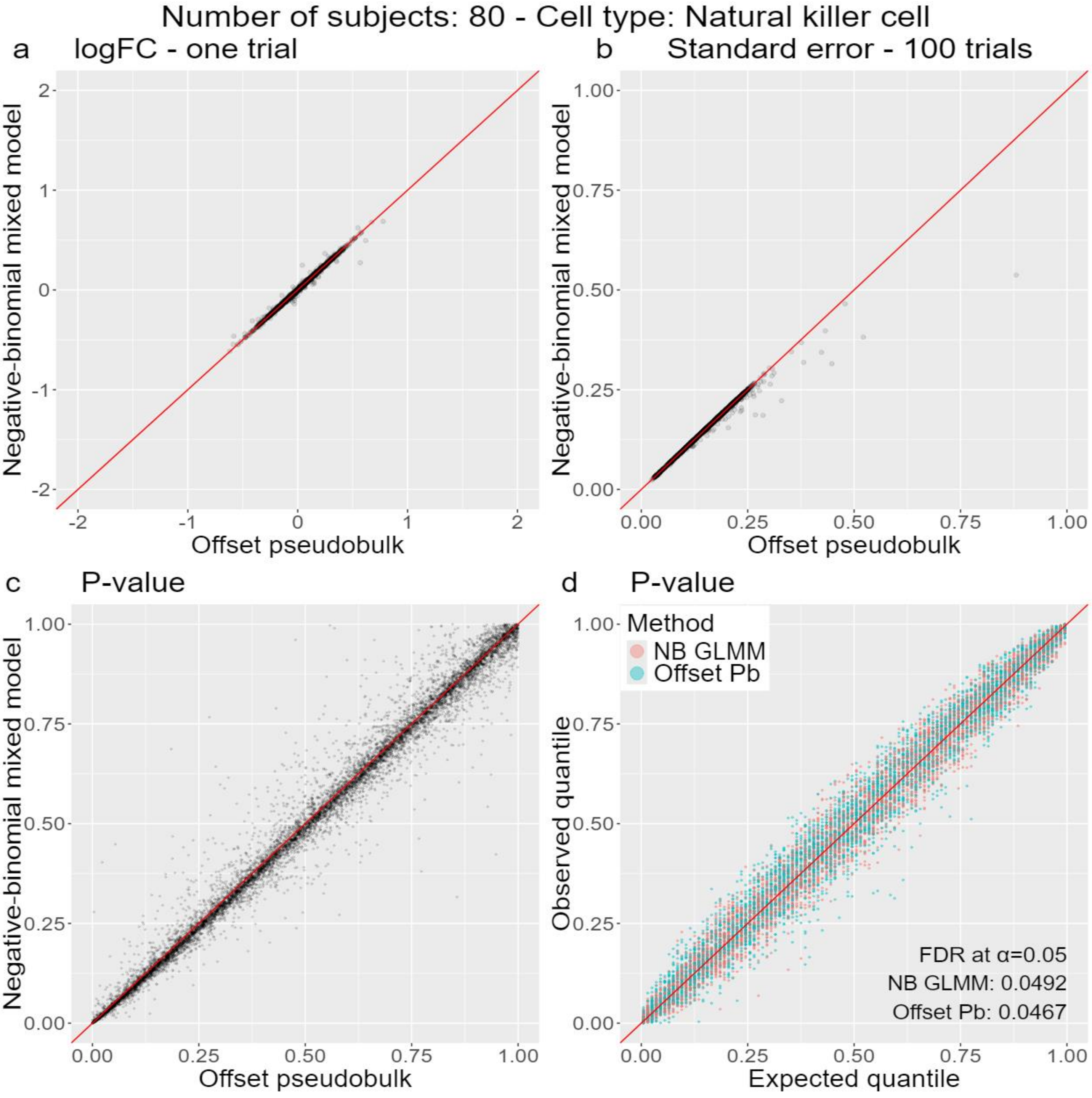

Supplementary Figure 18 a. Point estimates of the two methods in one of the 100 trials. **b.** Standard error of two methods computed from 100 trials. **c.** *P*-values of the two methods in 100 trials across transcripts with a mean above 0.1. **d.** *P*-values of the two methods plotted against the expected distribution across 100 trials.

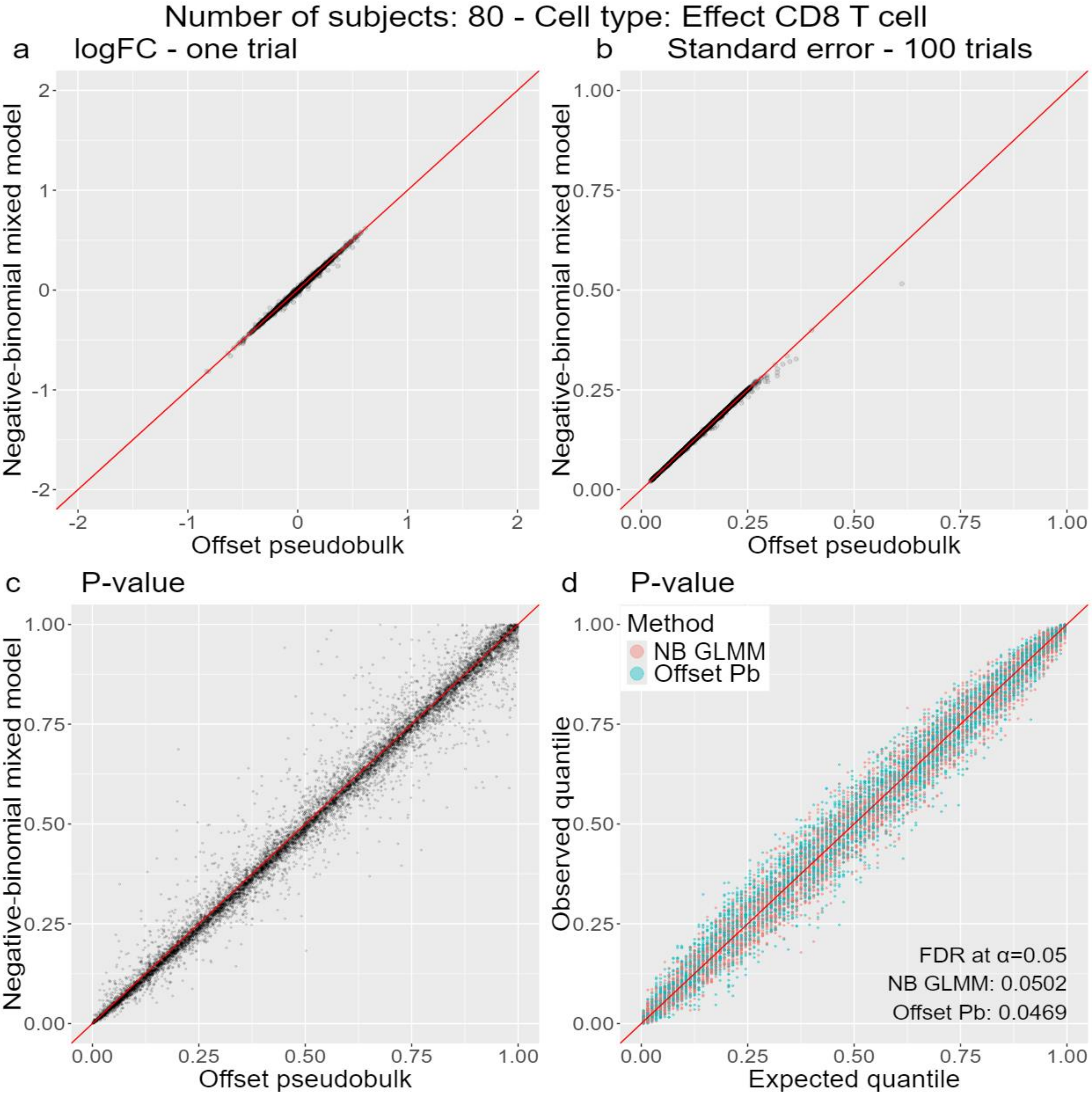

Supplementary Figure 19 a. Point estimates of the two methods in one of the 100 trials. **b.** Standard error of two methods computed from 100 trials. **c.** *P*-values of the two methods in 100 trials across transcripts with a mean above 0.1. **d.** *P*-values of the two methods plotted against the expected distribution across 100 trials.

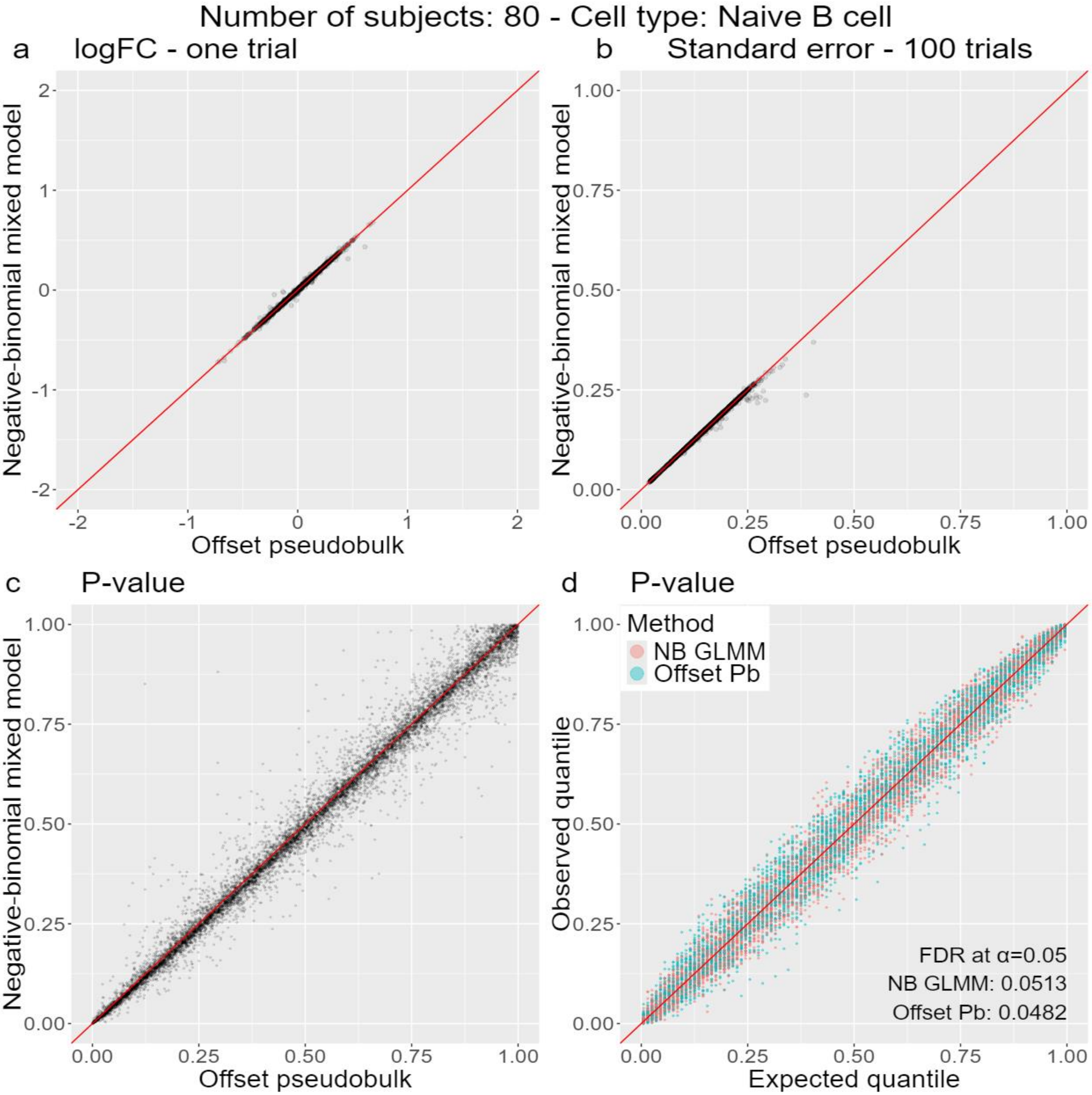

Supplementary Figure 20 a. Point estimates of the two methods in one of the 100 trials. **b.** Standard error of two methods computed from 100 trials. **c.** *P*-values of the two methods in 100 trials across transcripts with a mean above 0.1. **d.** *P*-values of the two methods plotted against the expected distribution across 100 trials.

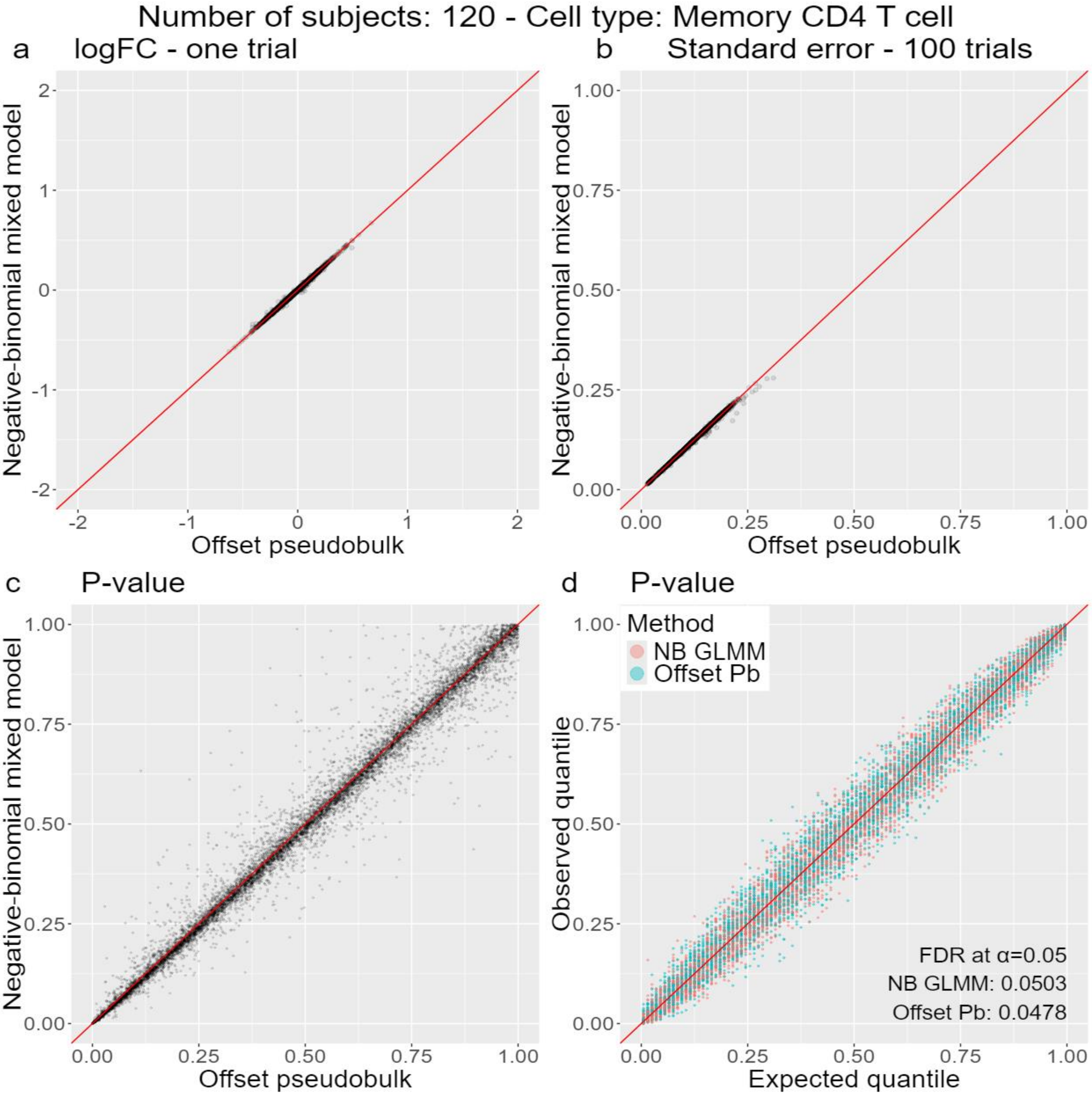

Supplementary Figure 21 a. Point estimates of the two methods in one of the 100 trials. **b.** Standard error of two methods computed from 100 trials. **c.** *P*-values of the two methods in 100 trials across transcripts with a mean above 0.1. **d.** *P*-values of the two methods plotted against the expected distribution across 100 trials.

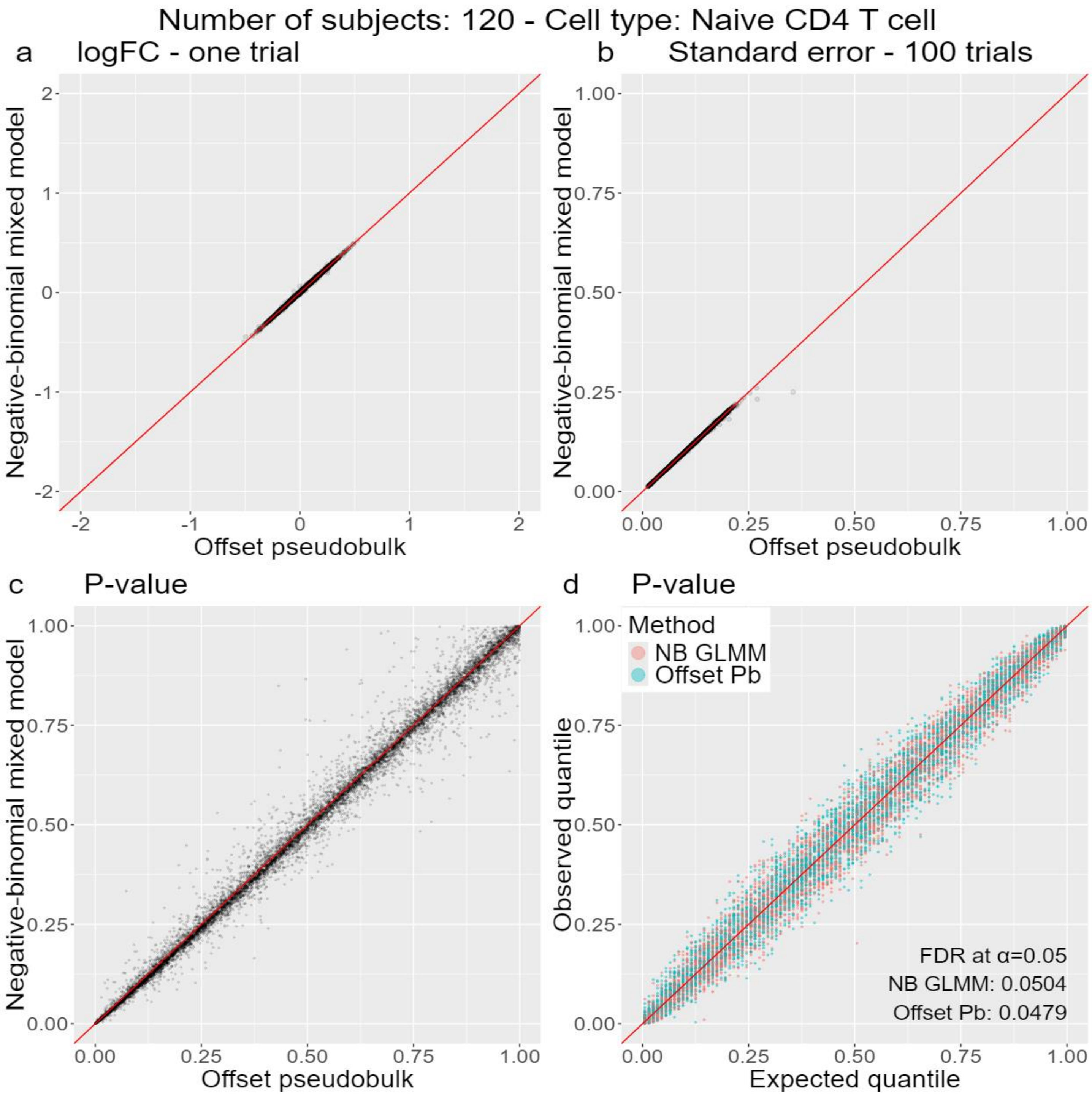

Supplementary Figure 22 a. Point estimates of the two methods in one of the 100 trials. **b.** Standard error of two methods computed from 100 trials. **c.** *P*-values of the two methods in 100 trials across transcripts with a mean above 0.1. **d.** *P*-values of the two methods plotted against the expected distribution across 100 trials.

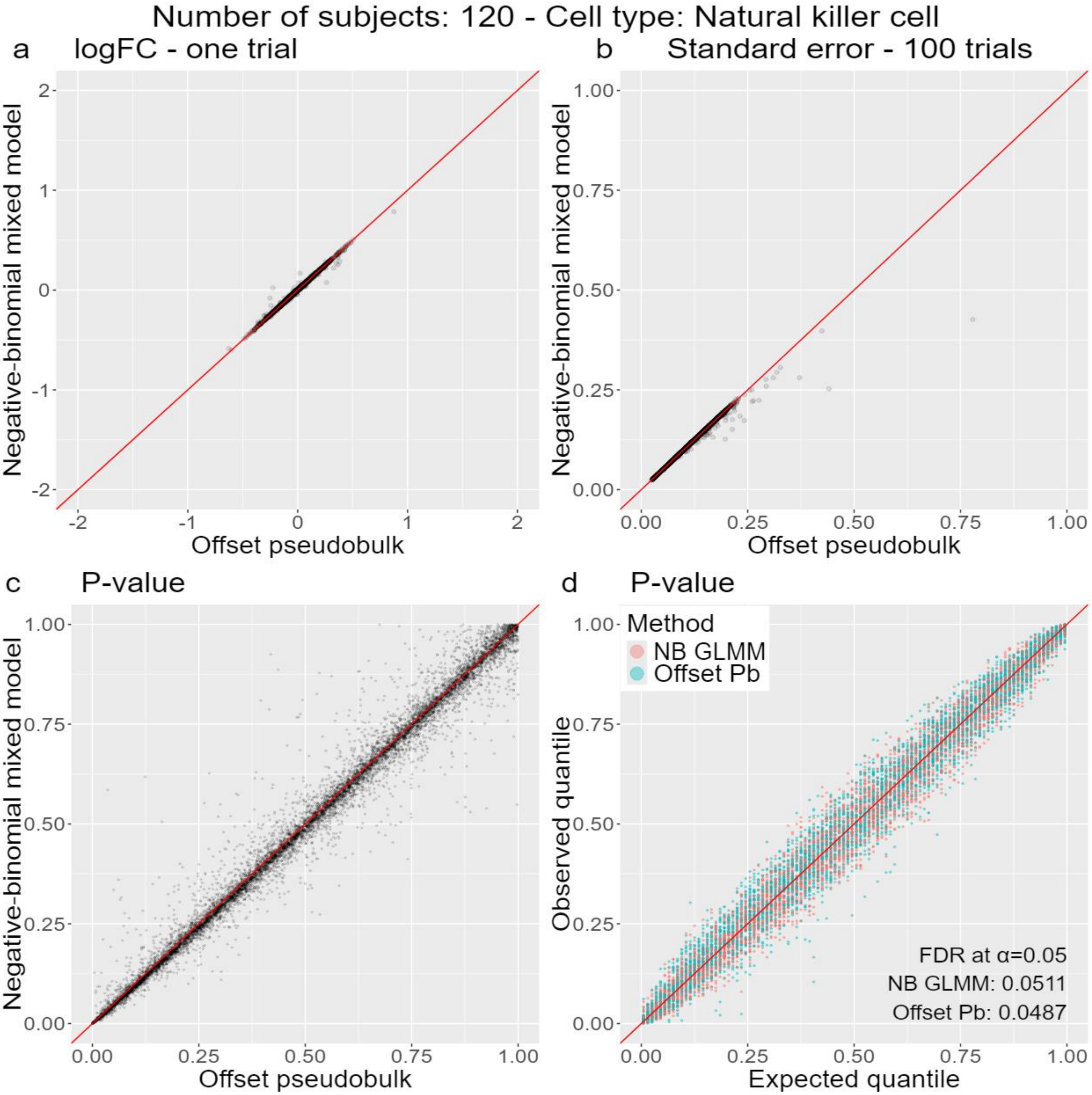

Supplementary Figure 23 a. Point estimates of the two methods in one of the 100 trials. **b.** Standard error of two methods computed from 100 trials. **c.** *P*-values of the two methods in 100 trials across transcripts with a mean above 0.1. **d.** *P*-values of the two methods plotted against the expected distribution across 100 trials.

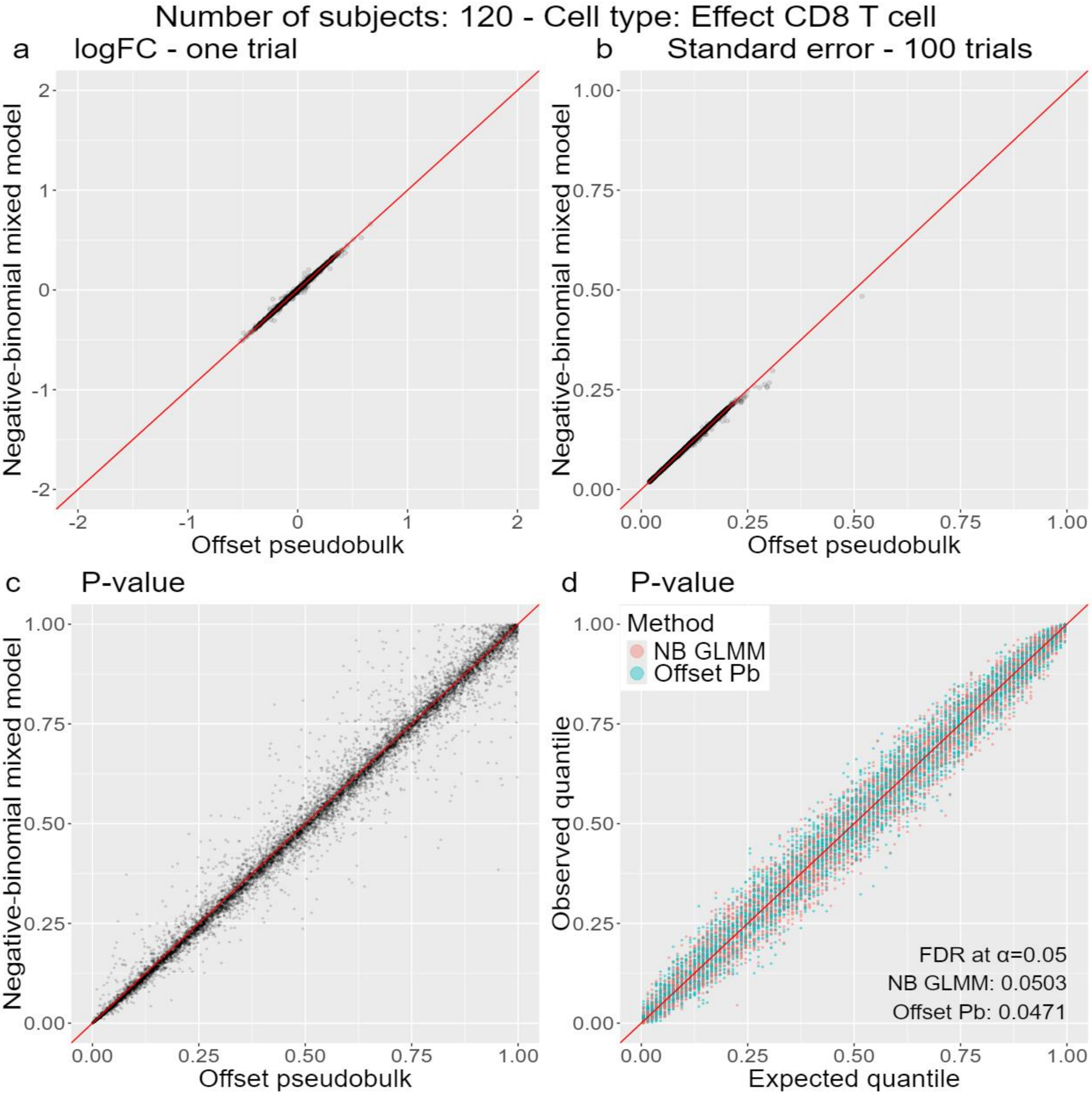

Supplementary Figure 24 a. Point estimates of the two methods in one of the 100 trials. **b.** Standard error of two methods computed from 100 trials. **c.** *P*-values of the two methods in 100 trials across transcripts with a mean above 0.1. **d.** *P*-values of the two methods plotted against the expected distribution across 100 trials.

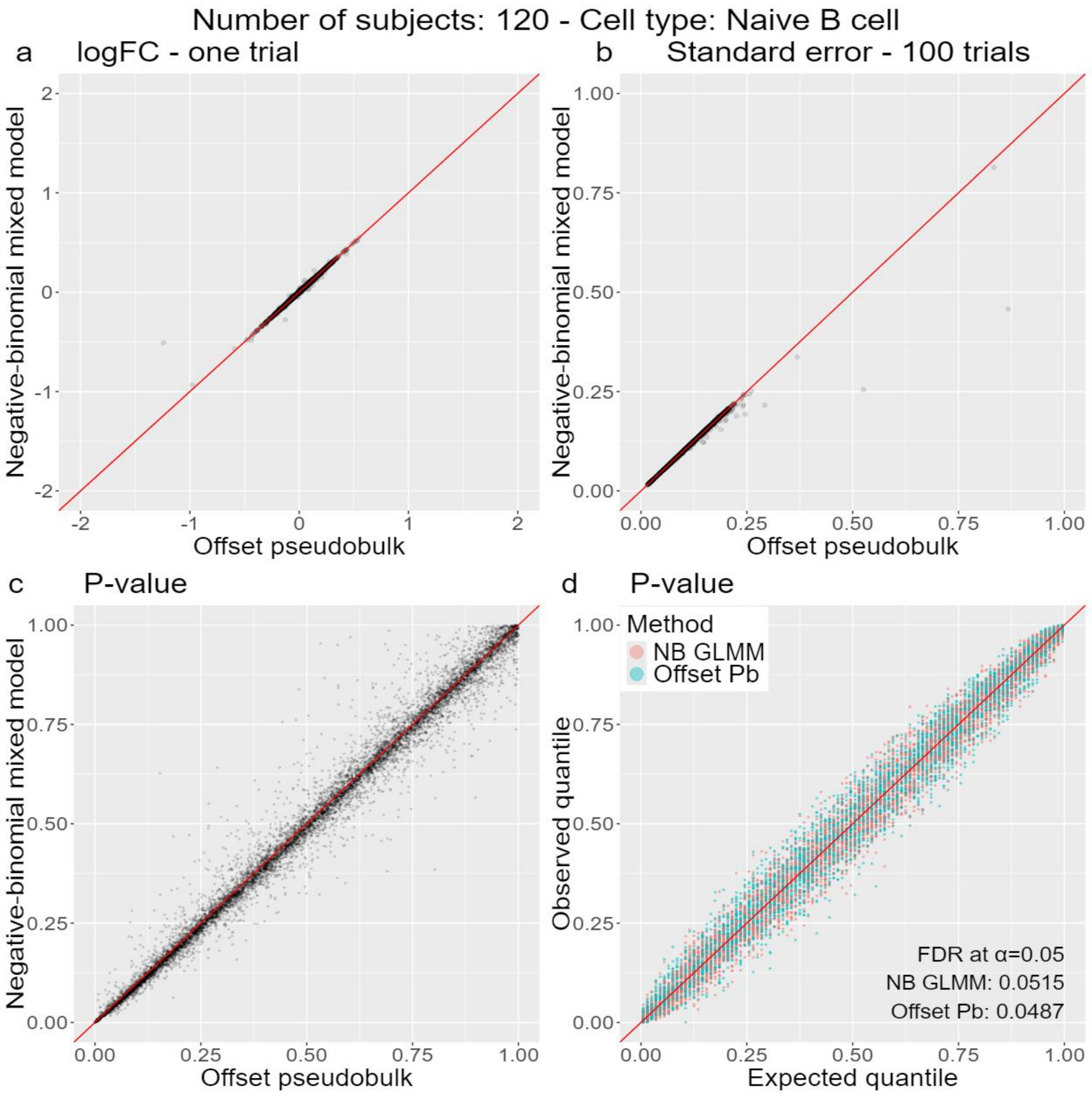

Supplementary Figure 25 a. Point estimates of the two methods in one of the 100 trials. **b.** Standard error of two methods computed from 100 trials. **c.** *P*-values of the two methods in 100 trials across transcripts with a mean above 0.1. **d.** *P*-values of the two methods plotted against the expected distribution across 100 trials.

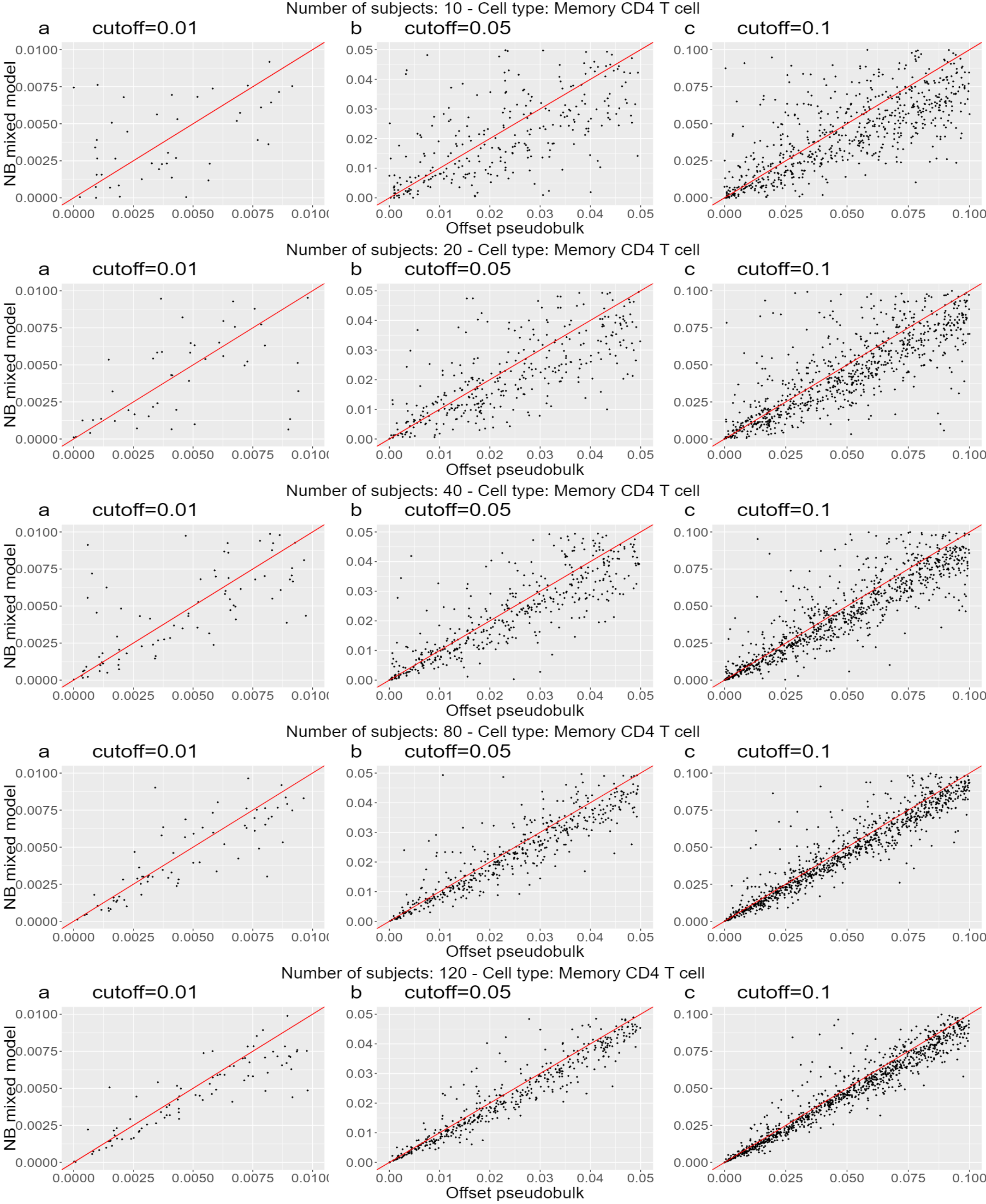

Supplementary Figure 26 Zoomed-in figures of **Figure 1c** and corresponding supplementary figures. Memory CD4 T cell

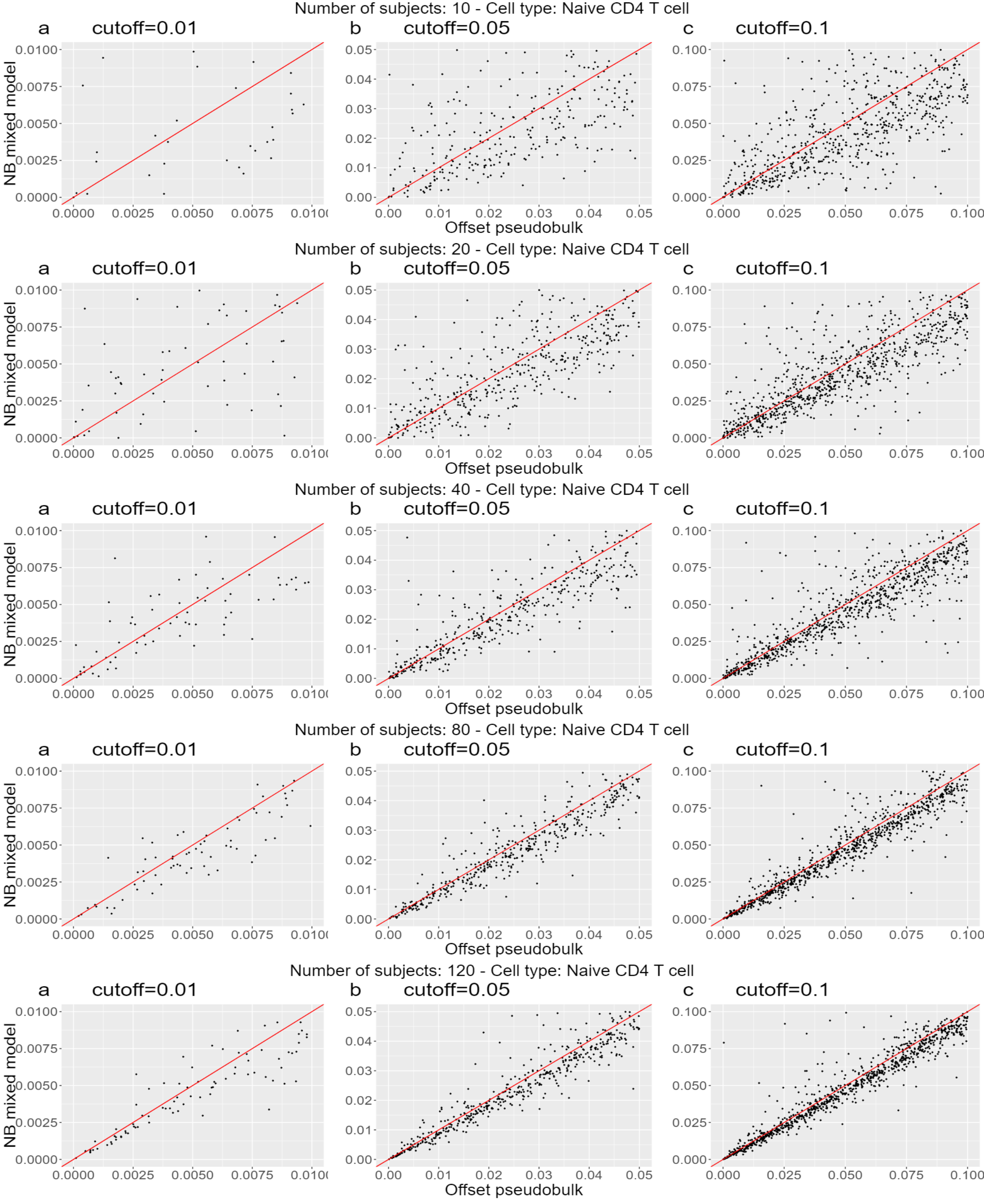

Supplementary Figure 27 Zoomed-in figures of **Figure 1c** and corresponding supplementary figures. Naïve CD4 T cell

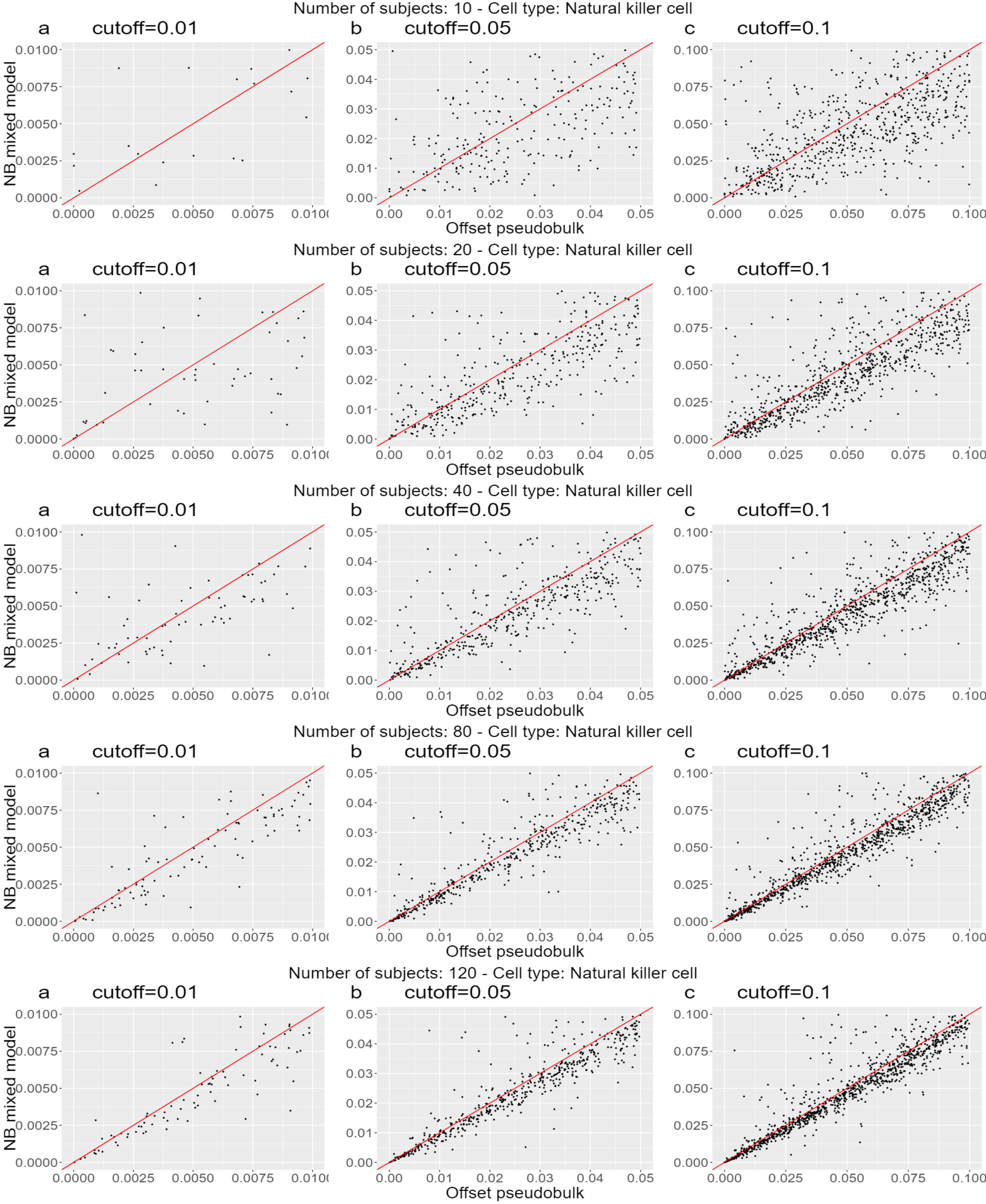

Supplementary Figure 28 Zoomed-in figures of **Figure 1c** and corresponding supplementary figures. Natural killer cell

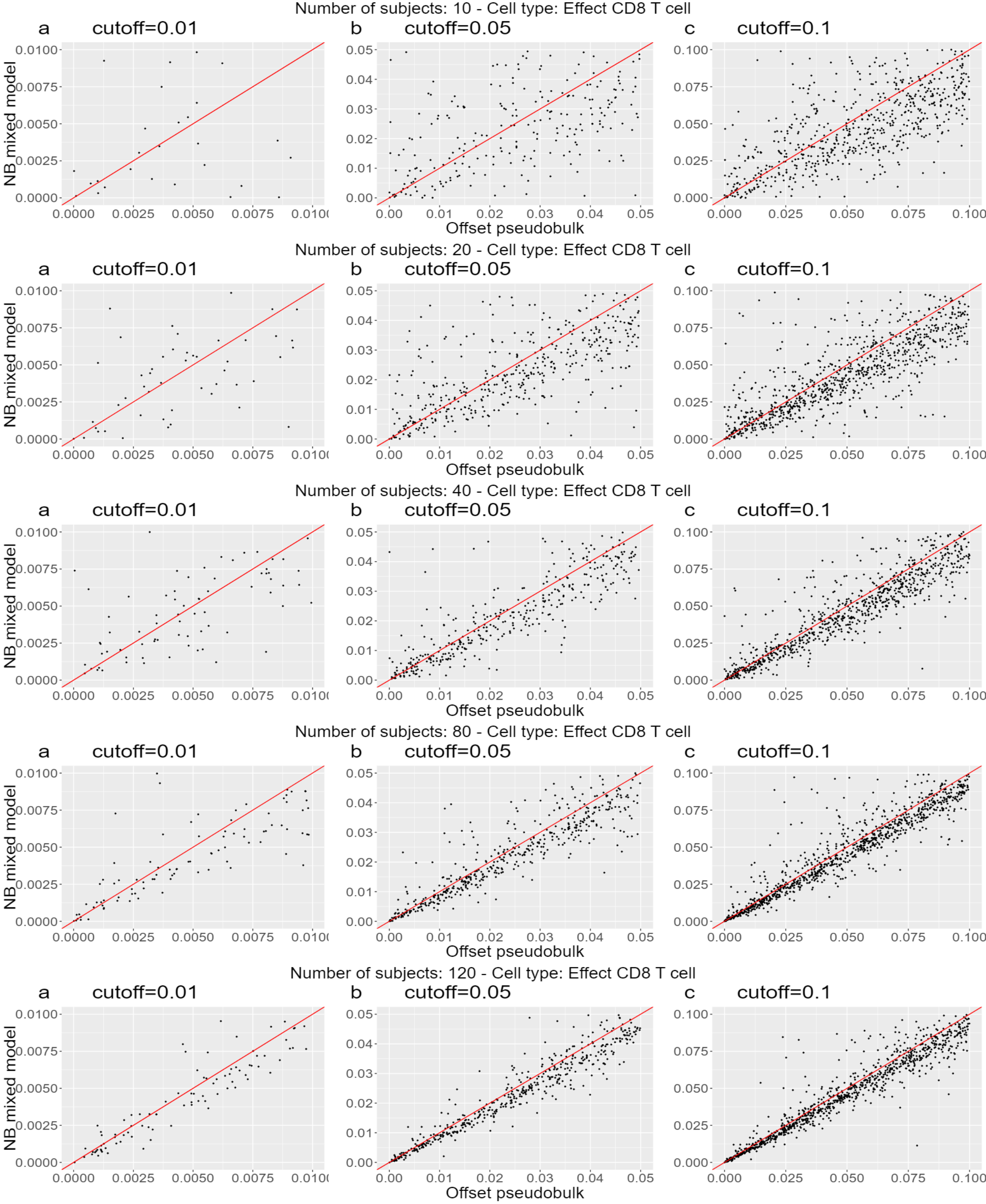

Supplementary Figure 29 Zoomed-in figures of **Figure 1c** and corresponding supplementary figures. Effect CD8 T cell

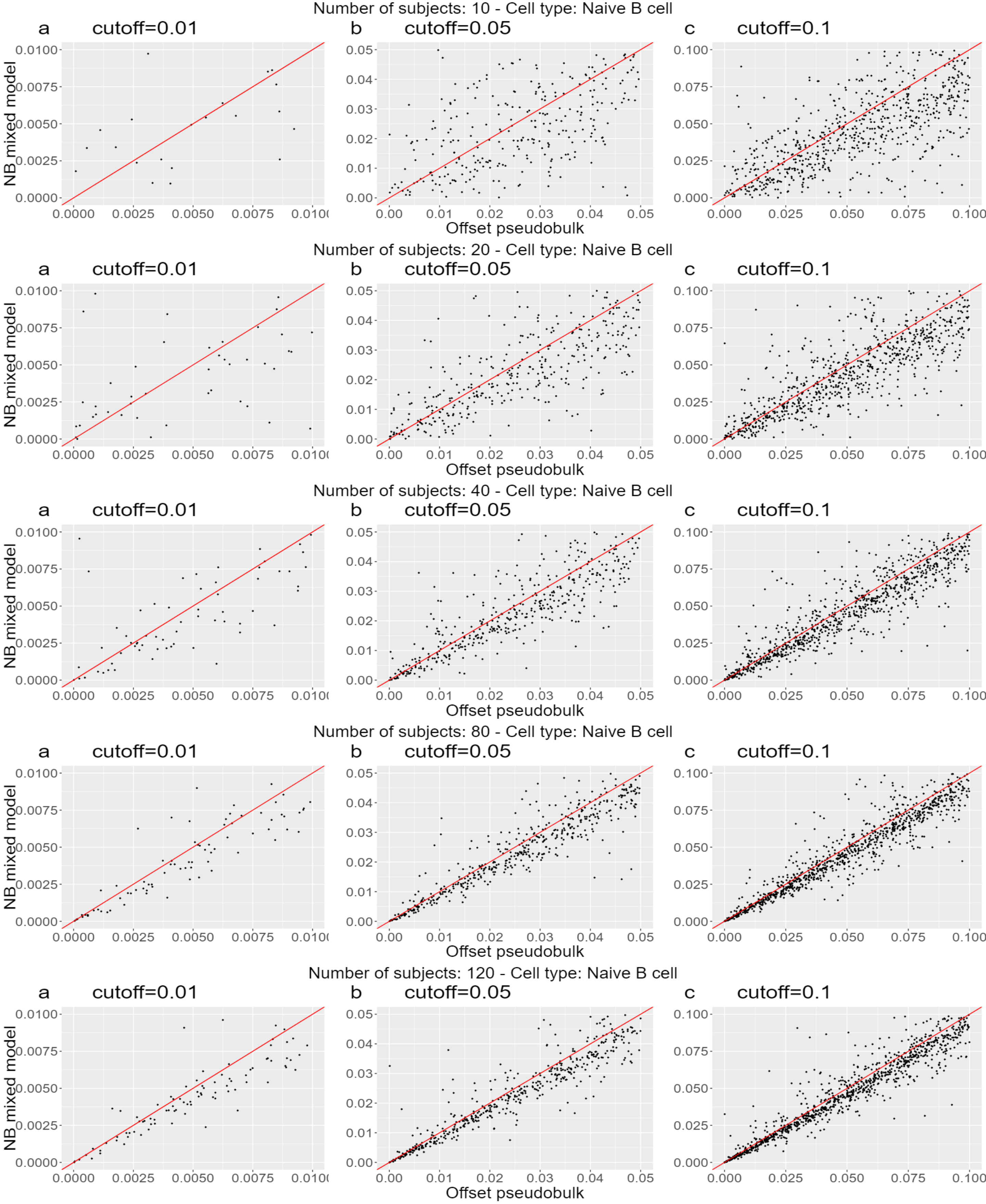

Supplementary Figure 30 Zoomed-in figures of **Figure 1c** and corresponding supplementary figures. Naïve B cell

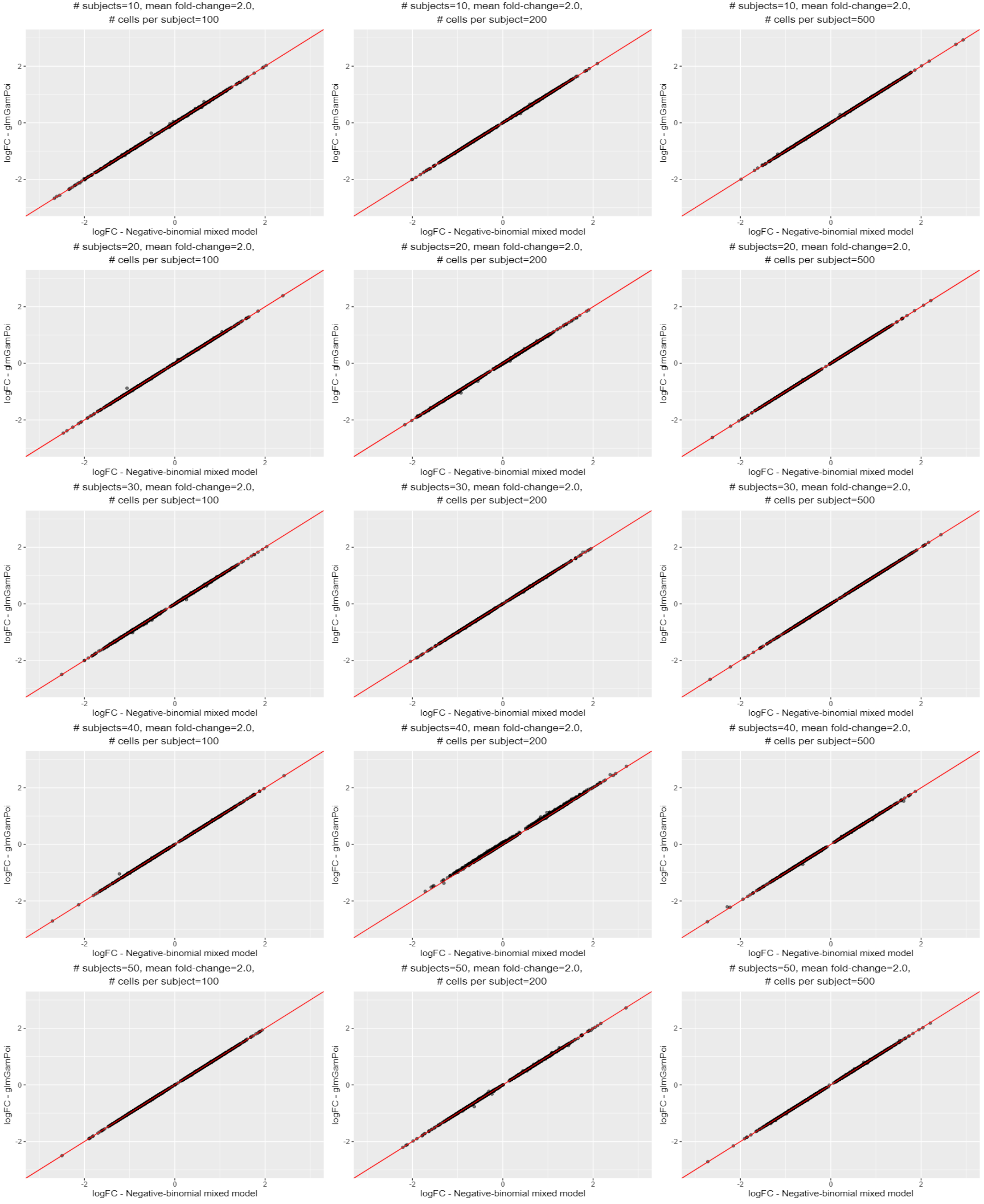

Supplementary Figure 31 Comparison of glmGamPoi and NEBULA point estimates across varying conditions in simulated data. Average fold change = 2.0

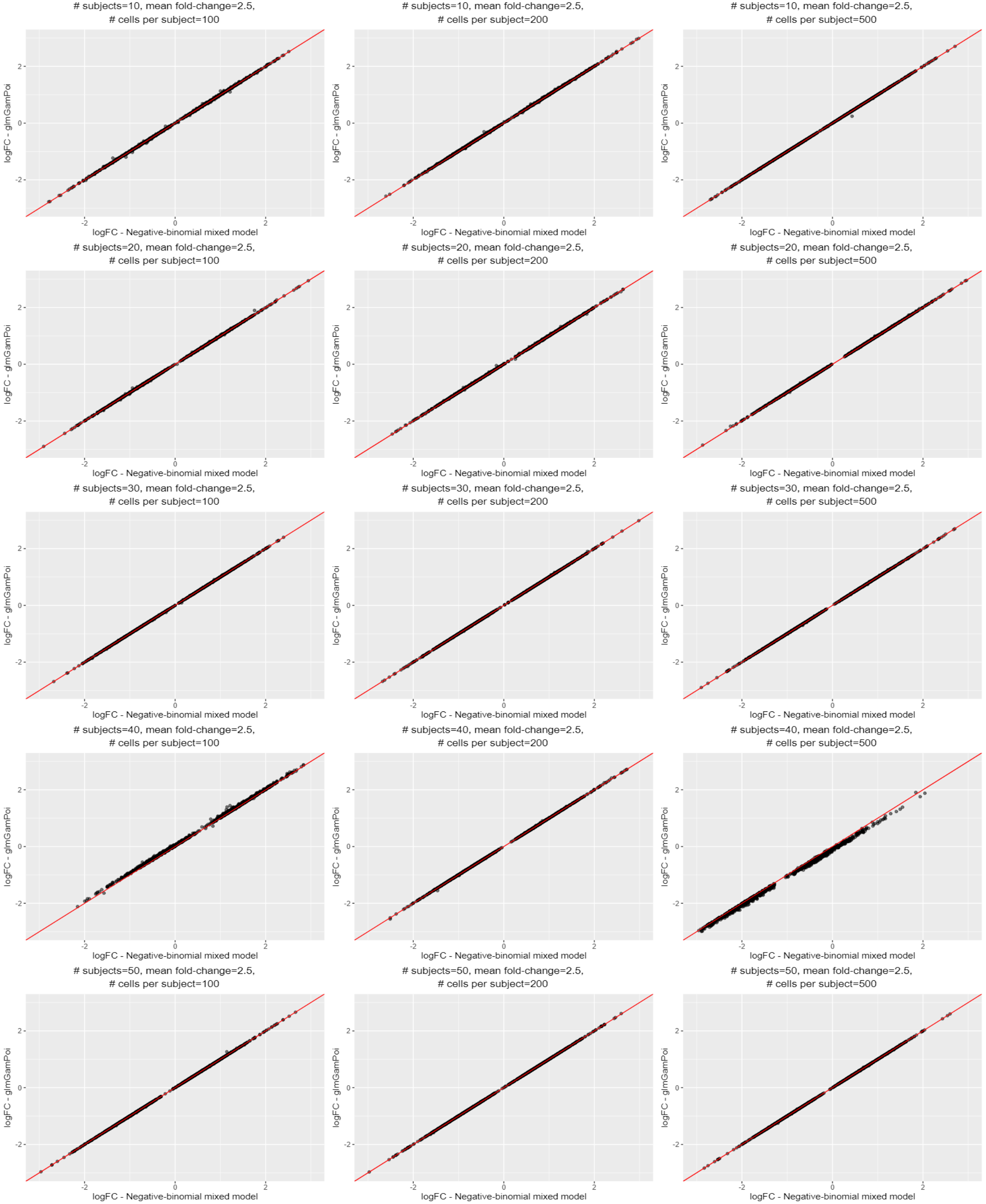

Supplementary Figure 32 Comparison of glmGamPoi and NEBULA point estimates across varying conditions in simulated data. Average fold change = 2.5

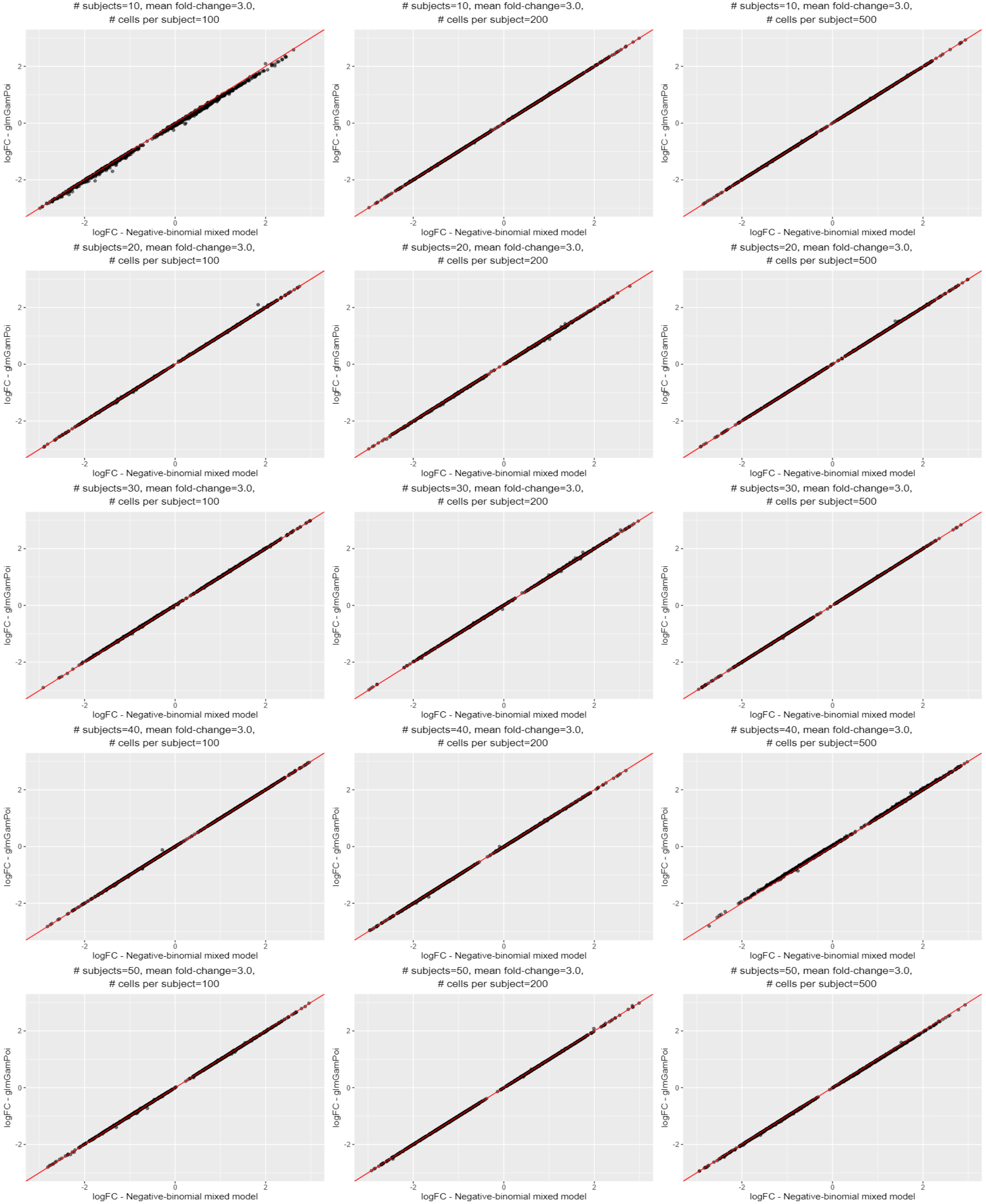

Supplementary Figure 33 Comparison of glmGamPoi and NEBULA point estimates across varying conditions in simulated data. Average fold change = 3.0

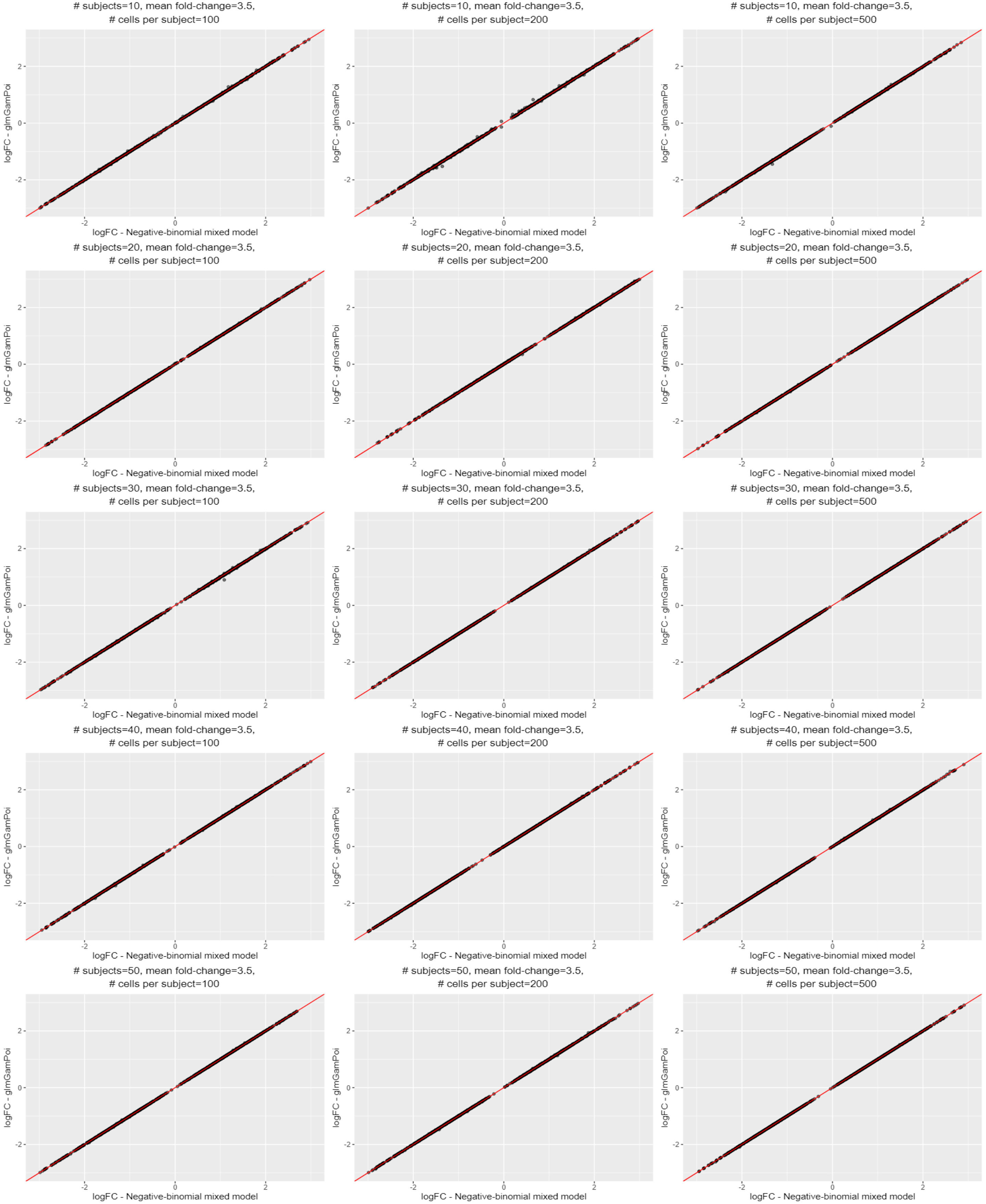

Supplementary Figure 34 Comparison of glmGamPoi and NEBULA point estimates across varying conditions in simulated data. Average fold change = 3.5

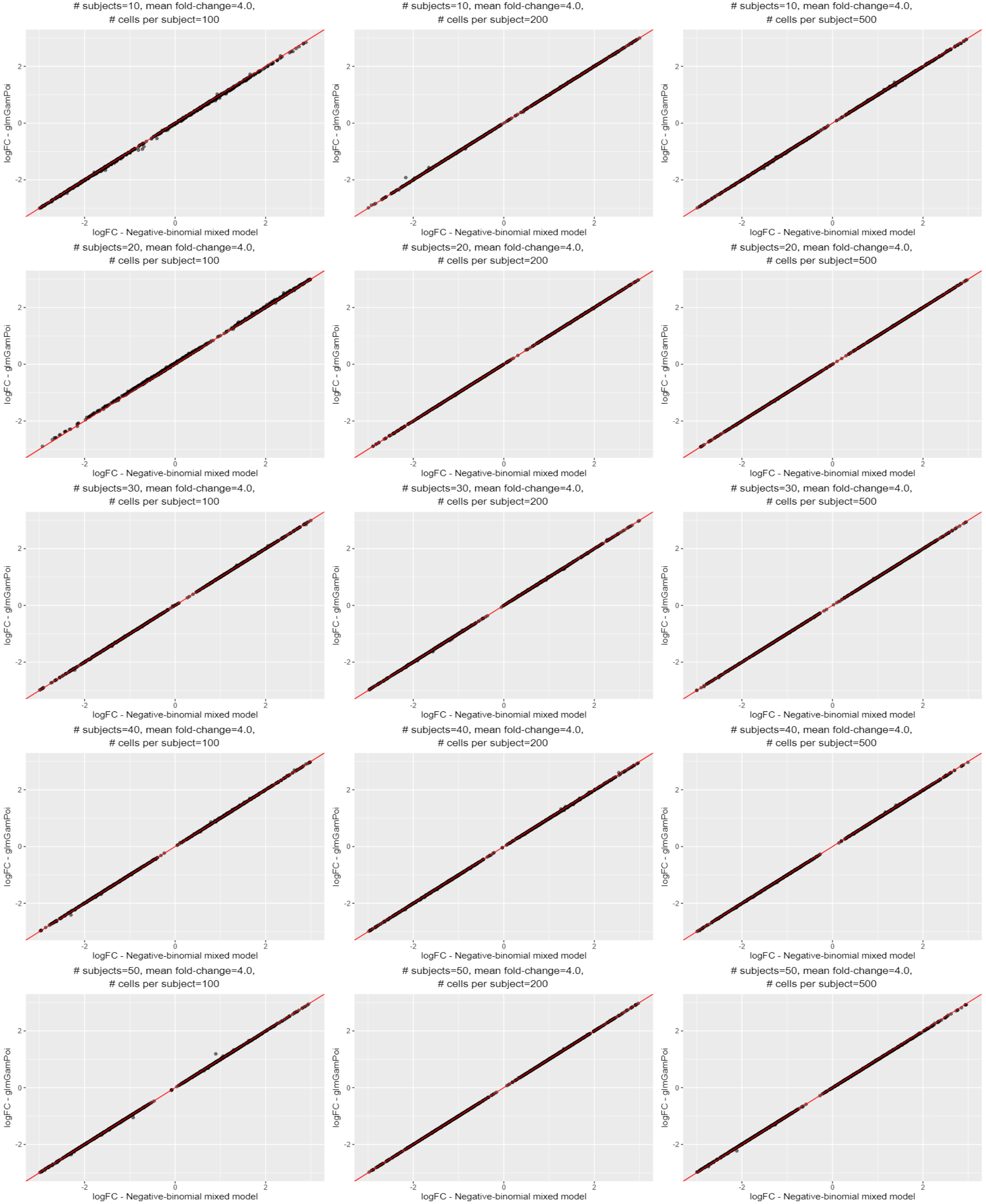

Supplementary Figure 35 Comparison of glmGamPoi and NEBULA point estimates across varying conditions in simulated data. Average fold change = 4.0

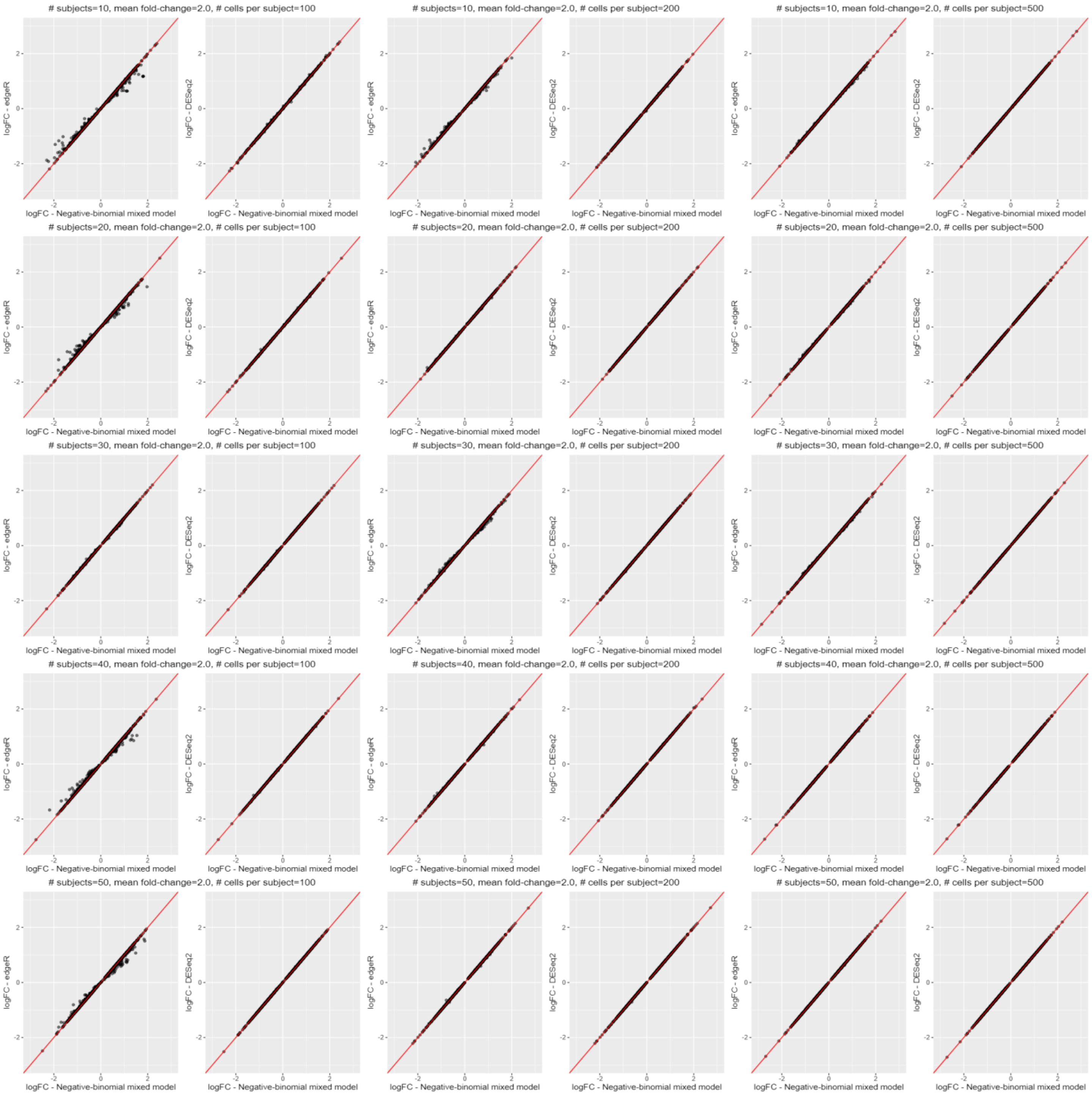

Supplementary Figure 36 Comparison of DESeq2/edgeR and NEBULA point estimates across varying conditions in simulated data. Average fold change = 2.0

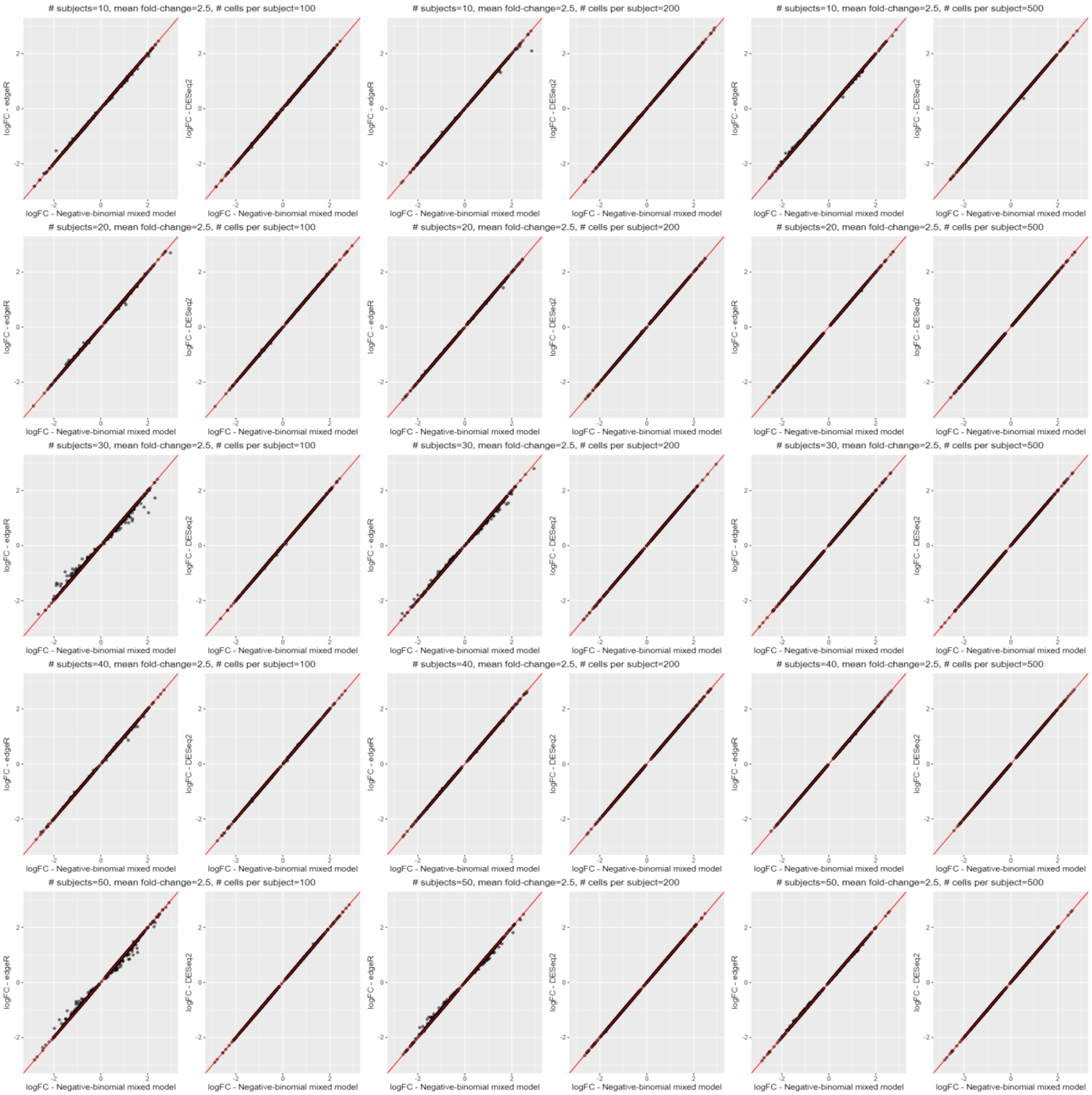

Supplementary Figure 37 Comparison of DESeq2/edgeR and NEBULA point estimates across varying conditions in simulated data. Average fold change = 2.5

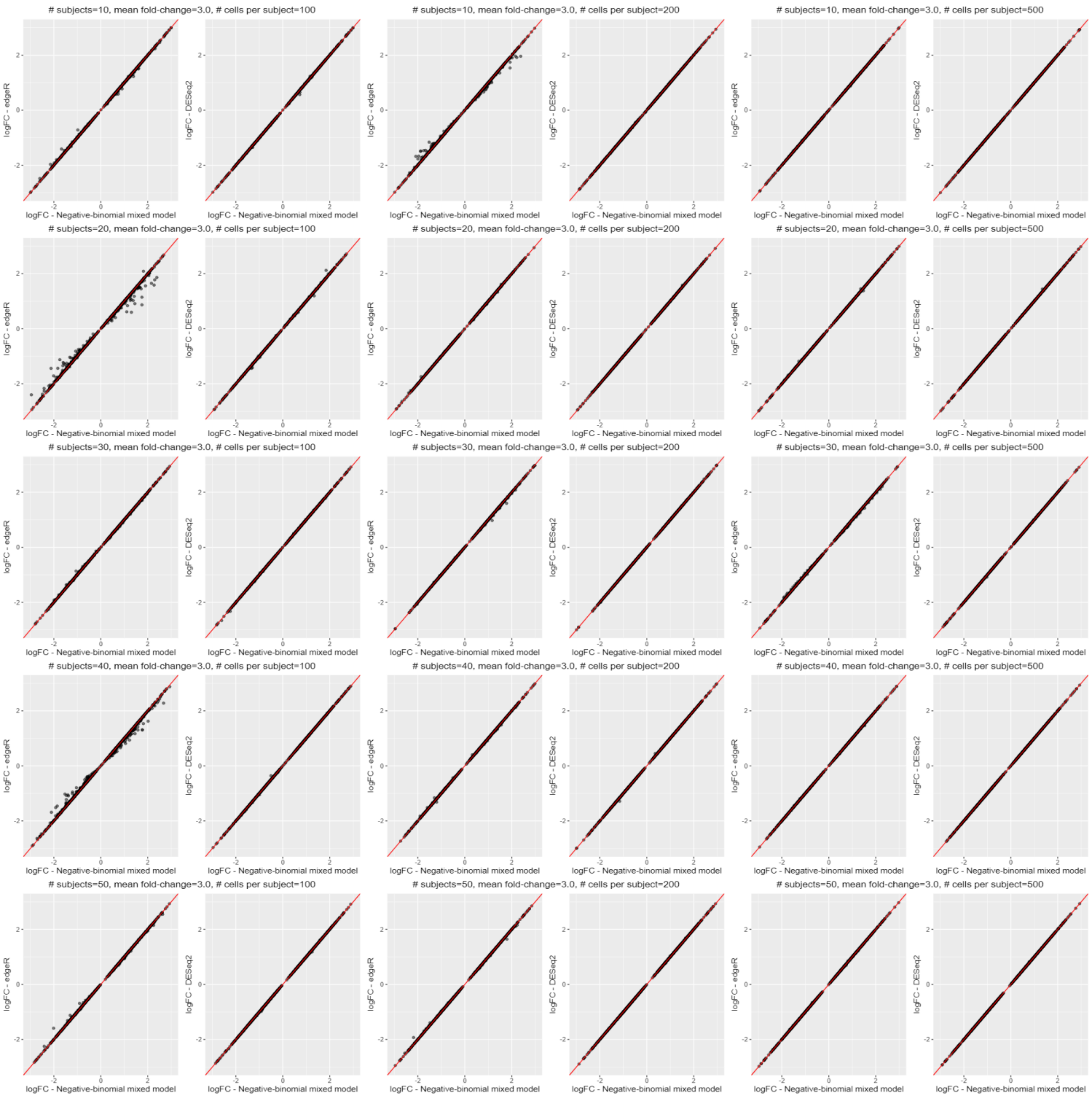

Supplementary Figure 38 Comparison of DESeq2/edgeR and NEBULA point estimates across varying conditions in simulated data. Average fold change = 3.0

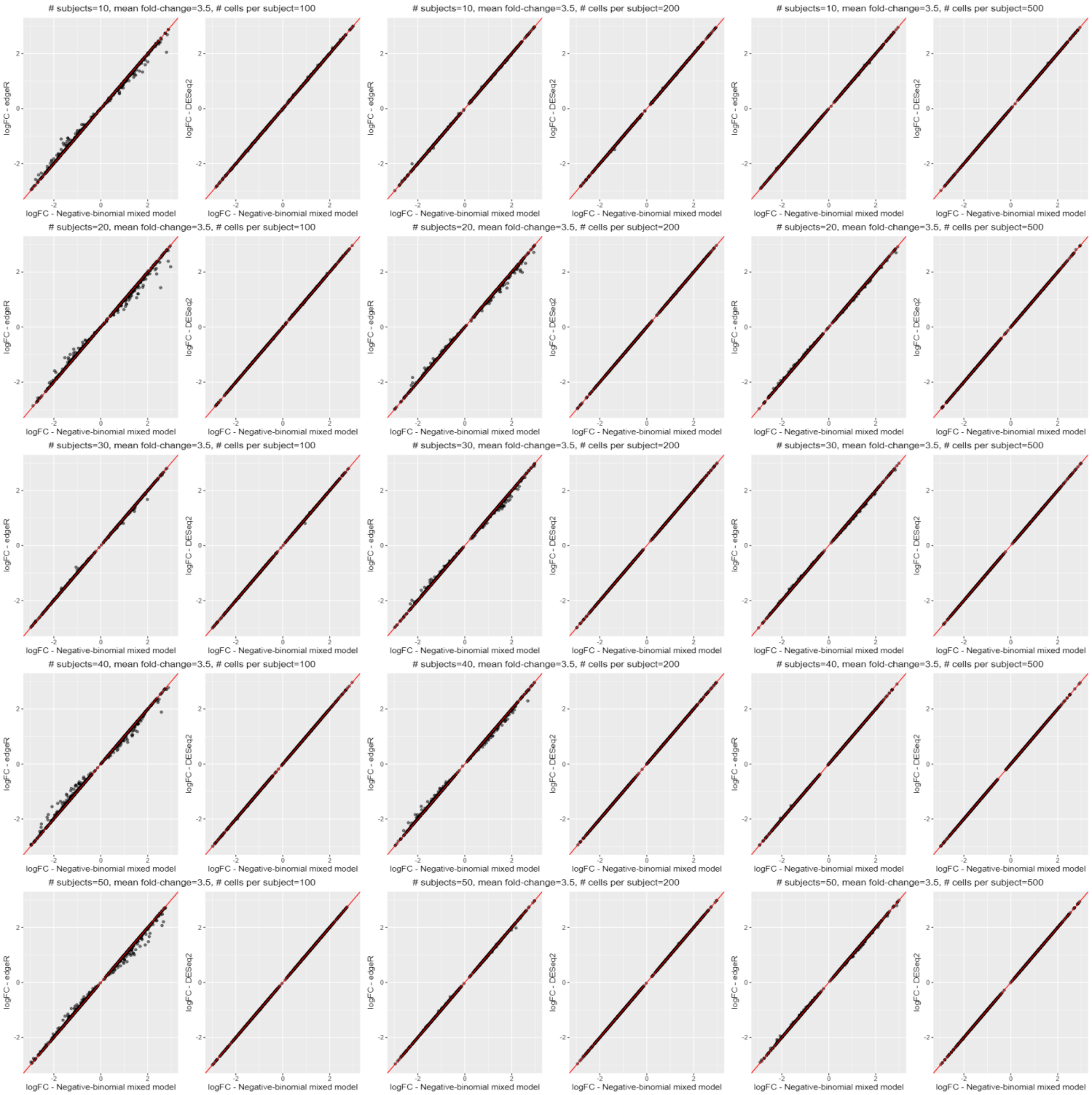

Supplementary Figure 39 Comparison of DESeq2/edgeR and NEBULA point estimates across varying conditions in simulated data. Average fold change = 3.5

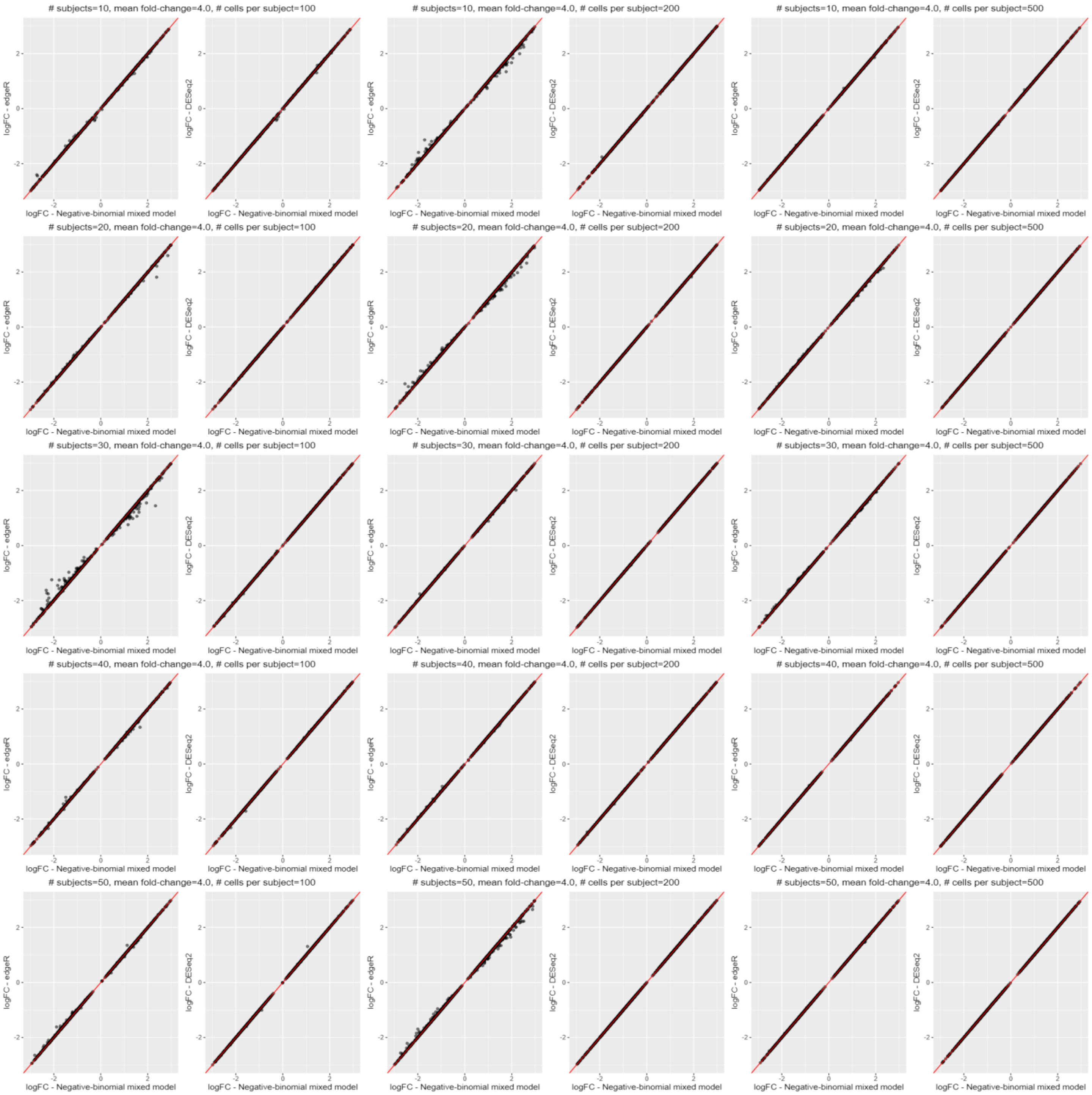

Supplementary Figure 40 Comparison of DESeq2/edgeR and NEBULA point estimates across varying conditions in simulated data. Average fold change = 4.0

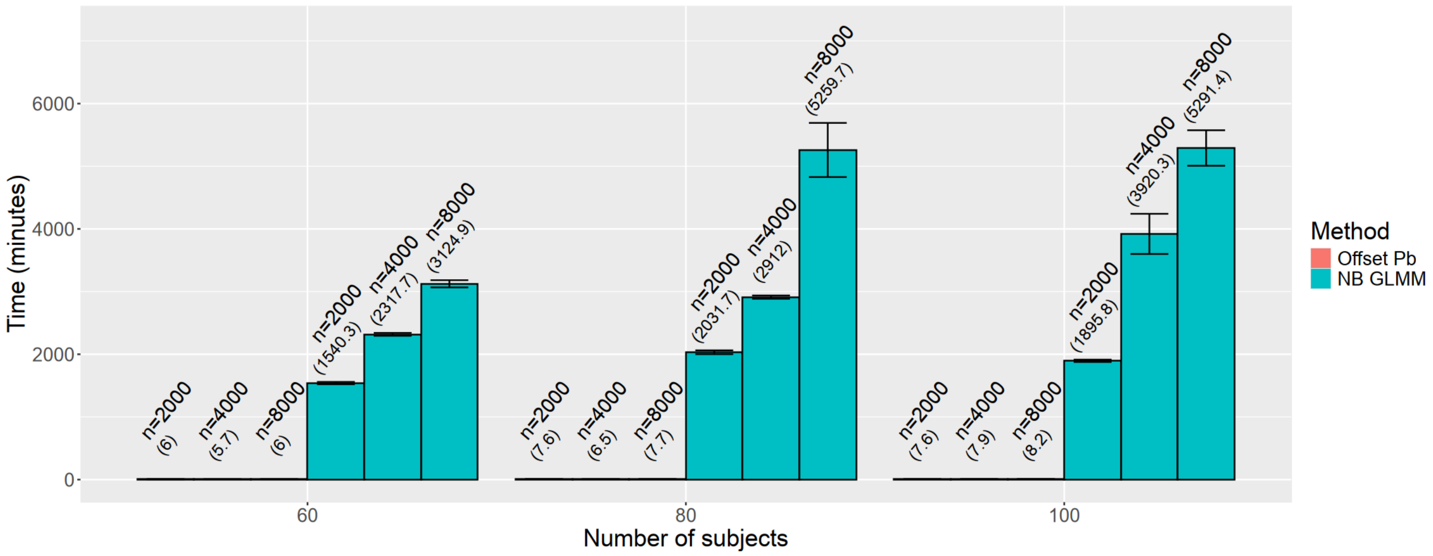

Supplementary Figure 41 Runtime comparison of glmGamPoi and NEBULA on simulated data. The tilted *n* indicates the number of cells per subject, and the number in the parentheses is the runtime.
